# Isolation, antibacterial screening, and identification of bioactive cave dwelling bacteria in Fiji

**DOI:** 10.3389/fmicb.2022.1012867

**Published:** 2022-12-20

**Authors:** Atanas Pipite, Peter J. Lockhart, Patricia A. McLenachan, Ketan Christi, Dinesh Kumar, Surendra Prasad, Ramesh Subramani

**Affiliations:** ^1^School of Agriculture, Geography, Environment, Ocean and Natural Sciences (SAGEONS), The University of the South Pacific, Suva, Fiji; ^2^School of Natural Sciences, Massey University, Palmerston North, New Zealand

**Keywords:** bioactive bacteria, actinomycetes, caves, antibiotics, selective isolation, bioactivity screening, 16S rRNA gene, Fiji

## Abstract

Bacteria are well known producers of bioactive secondary metabolites, including some of the most effective antibiotics in use today. While the caves of Oceania are still largely under-explored, they form oligotrophic and extreme environments that are a promising source for identifying novel species of bacteria with biologically active compounds. By using selective media that mimicked a cave environment, and pretreatments that suppressed the growth of fast-growing bacteria, we have cultured genetically diverse bacteria from a limestone cave in Fiji. Partial 16S rRNA gene sequences from isolates were determined and compared with 16S rRNA gene sequences in EzBioCloud and SILVA data bases. Fifty-five isolates purified from culture had Actinomycete-like morphologies and these were investigated for antibacterial activity. Initial screening using a cross streak test with pathogenic bacteria indicated that 34 of the isolates had antibacterial properties. The best matches for the isolates are bacteria with potential uses in the manufacture of antibiotics and pesticides, in bioremediation of toxic waste, in biomining, in producing bioplastics, and in plant growth promotion. Nineteen bacteria were confirmed as Actinomycetes. Thirteen were from the genus *Streptomyces* and six from genera considered to be rare Actinomycetes from *Pseudonocardia, Kocuria, Micromonospora, Nonomuraea*. Ten isolates were Firmicutes from the genera *Bacillus, Lysinbacillus, Psychrobacillus* and *Fontibacillus*. Two were Proteobacteria from the genera *Mesorhizobium* and *Cupriavidus*. Our findings identify a potentially rich source of microbes for applications in biotechnologies.

## Introduction

For thousands of years, yeasts, molds, and bacteria have been used to produce food, and for more than 7 decades, microbes have provided a valuable source of bioactive natural products important for the pharmaceutical industry (Sekurova et al., 2019). The need to find ecofriendly strategies for agricultural production has also led to the search for plant growth promoting micro-organisms with the potential to ameliorate abiotic stress, improve the uptake of mineral nutrients from soils, and act as biocontrol agents (Menéndez et al., 2020; Koskey et al., 2021). These different biotechnology applications put microbe’s center stage for the sustainable development of human societies. They perhaps receive greatest attention in the context of infectious disease. Motivated by the alarming rise of antimicrobial resistance (World Health Organization (WHO), 2015; World Health Organization (WHO), 2019), there has been renewed interest in the exploration of non-traditional environmental niches and novel methods for isolation of bacteria and their metabolites that may serve as antibiotics (Demain and Fang, 2000; Setlow, 2006; McMurry, 2015; Van Boeckel et al., 2015; Frère and Rigali, 2016; Thirumurugan et al., 2018; Abadi et al., 2019; Kadwalia et al., 2019; Liu et al., 2019; Beyer and Paulin, 2020; Litwin et al., 2020; Pantoja et al., 2020; Yehouenou et al., 2020; World Health Organization, 2020a,b).

While *Bacillus*, *Pseudomonas,* myxobacteria, and cyanobacteria are all known producers of antibiotics (Chandra and Kumar, 2017), the majority of antibiotics have been identified from phylum Actinomycetota (Actinobacteria or Actinomycetes, Subramani and Sipkema, 2019). Commonly called “antibiotic makers,” these bacteria have been the source for most of the natural compounds currently used in human therapies (Frère and Rigali, 2016; Thirumurugan et al., 2018). Two-thirds of known antibiotics are produced by the largest genus in Actinomycetota namely *Streptomyces* (Fernández-Martínez, 2019), but antibiotics have also been derived from less well characterized “rare” Actinomycetes (non-*Streptomyces* species). The number of antibiotics produced by rare Actinomycetes has increased by 25–30% over the last 2 decades due to improvement in techniques for selective isolation of rare-Actinomycetes and technologies for genetic characterization (Subramani and Aalbersberg, 2013; Axenov-Gribanov et al., 2016b; Subramani and Sipkema, 2019).

Phylum Actinomycetota is one of the largest in Domain Bacteria. As of December 2020, it comprises six classes, 32 orders, and 73 families.^1^ All Actinomycetes are filamentous unicellular Gram-positive bacteria with genomes of high GC content that are omnipresent in both terrestrial and aquatic environments (Barka et al., 2016). They form substrate and aerial mycelium. They reproduce by binary fission or by producing spores or conidia (Anandan et al., 2016). Actinomycetes include thermophilic species (45–65°C; Liu et al., 2020), acidophilic species (pH 3.5–6.5; Gonzalez et al., 2020), halophilic species (0.5–4 M salt; Matroodi et al., 2020), endophytic species (Zahra et al., 2020), symbionts (Pozzi et al., 2020), and endosymbionts (Goudjal et al., 2016). Some Actinomycetes produce enzyme inhibitors involved in the bioconversion, biocontrol, and degradation of hydrocarbons (Anandan et al., 2016). But more commonly, they are recognized for their production of natural bioactive compounds that provide defenses against pathogenic microorganisms (Grasso et al., 2016; Przybylska-Balcerek et al., 2019).

In the previous decade, rediscovery of the same metabolites in *Streptomyces* species led to a loss of interest by the pharmaceutical industry in bioprospecting for novel Actinomycetes (Sekurova et al., 2019). However, with the emergence of multidrug resistant pathogens (Nelson, 2003), together with the knowledge that most bacteria still remain uncultured and uncharacterized (Locey and Lennon, 2016; Hahn et al., 2019; Steen et al., 2019), there has been motivation for a renewed search of under-explored extreme environments where antibiotic producing bacteria might be found. Caves represent one form of extreme environment that have become the focus of recent investigations.

Several studies have now reported Actinomycetes with antimicrobial, and anticancer activities as the dominant phylum of bacteria present in caves (Nimaichand et al., 2015; Axenov-Gribanov et al., 2016a; Adam et al., 2018; Hamedi et al., 2019; Long et al., 2019; Wiseschart et al., 2019; Zada et al., 2021). Actinomycetes colonize a variety of cave microhabitats. These include water, ceilings, floors (Hamedi et al., 2019), moonmilk (a deposit of carbonate minerals on cave walls), speleothems (stalagmites and stalactites; Nimaichand et al., 2015; Axenov-Gribanov et al., 2016a; Tomczyk-Żak and Zielenkiewicz, 2016; Maciejewska et al., 2018) as well as soil and bat guano (Long et al., 2019). Consequently, caves have become an important hunting ground for novel Actinomycetes, and a potential source for the discovery of bioactive compounds (Rangseekaew and Pathom-aree, 2019).

Caves are unusual environments characterized by scarcity of light, temperature, and nutrients (Cheeptham, 2013). Caves in particular that have little energy exchange with the external environment are of great scientific importance because they provide stable environments for the growth and selection of nutrient limited macro-and micro-organisms, including rare Actinomycetes (Kambesis, 2007; Hamedi et al., 2019). Each type of cave has specific properties depending on its heterogeneity of mineral and organic surfaces, and surrounding vegetation. Factors that affect microbial communities include pH, availability of nutrients, total organic carbon (TOC)/liter, low light, temperature, humidity, and geological and biotic histories (Cuezva et al., 2009; Bontemps et al., 2021; Iquebal et al., 2021; Prescott et al., 2022).

In the last decade, researchers have documented high microbial diversity in caves, including oceanic island caves (Northup et al., 2011; Wall et al., 2015; Prescott et al., 2022). However, to date, there appears to be no reports of bioactive bacteria isolated from the Island caves of Oceania. Oceania is a region made up of thousands of islands in the central and southern Pacific Ocean. Many of these caves are oligotrophic and tropical. They are a potential source for the discovery of bioactive bacteria that have evolved in response to the unique characteristics of low nutrient tropical ecosystems (Cuezva et al., 2009; Yasir, 2018; Sekurova et al., 2019).

We undertook a culture-based (culture-dependent) study to investigate bacteria with Actinomycete-like morphologies from a limestone cave in Fiji. A challenge for culture-based studies is finding culture conditions suitable for growing different species of bacteria. Those that have been cultured represent only a small proportion (0.1–10%) of the total bacteria present in the soil and rhizosphere (Wei et al., 2021). Furthermore, Actinomycetes have relatively slow growth rates (Anandan et al., 2016), and other fast-growing bacteria and fungi isolated from the same environment can outgrow them in culture. This makes their isolation difficult under laboratory conditions (Jiang et al., 2016). To overcome this problem, targeted selective isolation methods and strategies have been devised. One current strategy is to pretreat environmental samples to remove fast-growing bacteria and fungi before plating the diluted samples on selective growth media (Subramani and Aalbersberg, 2013). Actinomycete spores are very resistant to harsh chemicals, heat, and extreme drought conditions. In addition, Actinomycetes can germinate under conditions unfavorable for the survival of other bacteria. By selecting against unwanted microbes, and providing conditions suitable for Actinomycetes, pretreatments have been found to significantly increase the number of Actinomycetes that can be recovered (Tiwari and Gupta, 2012; Subramani and Aalbersberg, 2013; Jiang et al., 2016).

In the present study, we made a collection of soil, stalactites, moonmilk, and bat guano and cultured bacteria from these samples. These cultures were screened for bioactivity and cultures have been characterized using16S rRNA database comparisons and phylogenetic analyses. Our findings provide first insights into the high diversity of bioactive bacteria, including Actinomycetes residing in the caves of Fiji.

## Materials and methods

### Sample collection

Permission to visit the cave system in Fiji was obtained by presenting a Sevusevu (traditional ceremony) to the chief and leaders of the community. The limestone cave sampled was home to a colony of Fijian bats (*Notopteris macdonaldi*) and the cave has various chambers and entrances, three of which were recorded by early European surveyors (Gilbert, 1984). To investigate the cave, its microbial diversity and antimicrobial potential, we surveyed the main chambers (western side) for our sample collection. The forest cover around the cave mouth appeared to be largely intact. The cave interior was completely dark with a light measurement of zero lux and a relative humidity of 70.5%. Temperature of sampling locations in the cave was measured using a digital thermometer during the same day of sample collection. Recorded temperatures of sampling locations varied from 23.5 to 25°C. The salinity of samples was measured within 24 h after collection with a hydrometer according to manufacturer’s instructions and the recorded values were approximately 3–4%. The pH of cave samples was also measured using universal pH indicator, and the pH range of samples ranged from 4.98 to 7.45. The results obtained for physiochemical parameters measured are listed in Supplementary Table S1. Samples for isolation of bacteria were collected from different part of the caves. The samples included moonmilk speleothems, bat guano, rock soil, and soil (Supplementary Figure S1). The samples were collected using sterile scalpels or spades and were transferred immediately into falcon tubes or ziploc bags. The samples were brought back to the laboratory as soon as possible, and then stored in the cold room at 4°C for about 6 h until use.

### Samples pretreatments and isolation of Actinomycetes

In the present study, four culture mediums and two pretreatments were used to culture Actinomycetes and other bacteria with bioactive potential. We have used four selective media in this study namely Actinomycetes Isolation Agar (AIA), International Streptomyces Agar 5 (ISP 5), Starch Casein Agar (SCA), and Humic Vitamin Agar (Supplementary Table S2).

Cave samples were either untreated, pretreated with phenol, or alternatively pretreated using wet heat, prior to inoculation onto four different selective media (Supplementary Table S2). Bacteria were isolated according to the standard serial dilution plating technique as described by Long et al. (2019). The concentration of NaCl in the media was adjusted to match the salinity measured in the cave. To inhibit the growth of fast-growing bacteria and fungi, all media were supplemented with cycloheximide (50 mg/L) and nalidixic acid (20 mg/L). The plates were incubated at 28°C for 14–30 days and colonies that displayed Actinomycete like morphologies were picked from the original isolation plates and then sub-cultured on the International Streptomyces Projects 2 (ISP 2) agar plates to recover pure cultures exhibiting uniform colony morphology. The purified cultures were maintained on ISP 2 medium slants at 4°C and stored in 25% (*v/v*) glycerol suspensions at −80°C.

### Screening of Actinomycetes for bioactive potential

**Figure 1:**
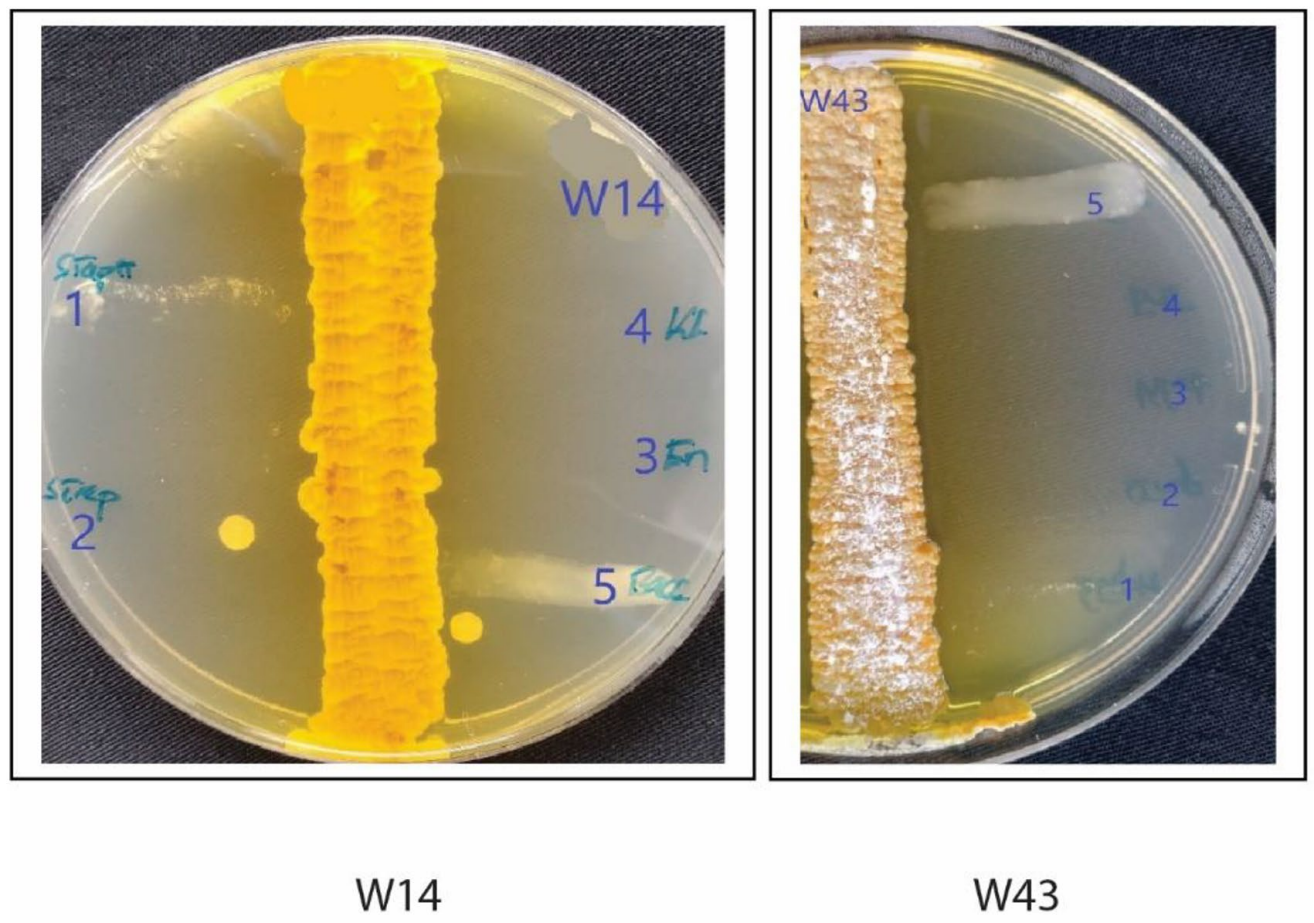
Cross-streak test results for cave isolates W14 and W43 showing zones of inhibition/antimicrobial activity, against five pathogenic bacterial strains (*Staphylococcus aureus*, *Streptococcus aureus*, *Enterococcus faecalis*, *Klebsiella pneumoniae*, and *Bacillus cereus*). Measurements (mm) for zones of inhibition for 34 isolates are given in Table 1. Supplementary Figure S2 illustrates the technique used for cross streaking.

Fifty-five isolates with Actinomycete-like morphology were selected for antimicrobial screening using a cross-streak method (Egorov, 1985). For each isolate, a single colony was streaked in a single straight line in the middle of a Petri dish containing International Streptomyces Agar 2 (ISP2; Figure 1; Supplementary Figure S2). After inoculation, the plates were incubated at 28°C for 10–14 days. Following the appearance of a well-defined ribbon of growth marking the original streak on the plates, freshly sub-cultured pathogen strains were streaked perpendicular to the original growth line of the cave isolate on the same plate. For sub-culturing, a single colony of each pathogenic bacteria was transferred to fresh nutrient broth and incubated at 37°C for 24 h until the visible turbidity and density equaled that of 0.5 McFarland [1.5 × 10^8 colony forming units (CFU/ml)]. After adjusting the turbidity, a sterile cotton swab was then dipped into the bacterial suspension and streaked perpendicular to the cave isolate on the agar medium. The streaked plates were incubated at 28°C for 24 h, and the strains producing antimicrobial compounds were selected by measuring their respective zones of inhibition measured in millimeters (mm). Zones of inhibition refer to areas where the pathogenic bacteria tested showed no growth on the streaked line. The indicator pathogens used for antimicrobial screening were *Staphylococcus aureus* (NZRM 917)*, Streptococcus pneumoniae, Klebsiella pneumonia, Enterococcus faecium* (NZRM 1106), and *Bacillus cereus*. Test pathogens were procured from the University of the South Pacific Biology laboratory, and originally sourced from Colonial War Memorial Hospital in Suva.

### DNA extraction and 16S rRNA gene PCR amplification from culture lysates

DNA lysates were prepared from single colonies that had grown on plates of selective media using the Microgem PrepGEM bacterial extraction kit (MicroGEM, New Zealand, https://microgembio.com/product/prepgem-bacteria-dna-extraction-kit/). The protocol is described below. The buffers are proprietary, and the kit has optional protocols for Gram-positive and Gram-negative bacterial extractions. A prewash step was included to remove polysaccharides, as Actinomycetes are known to produce them (Undabarrena et al., 2021). Actinomycetes are also gram-positive bacteria, so a lysozyme step was included to break down the cell wall (thick layer of peptidoglycan) of isolates. DNA extraction took place 7–14 days after plating which allowed sufficient time for the formation of aerial mycelia. Aerial mycelia from each isolate were transferred with a sterile loop into a 2 ml tube containing 0.4 ml of WASH+ buffer. The wash mixture was vortexed briefly to disperse the cells and centrifuged at 10,000 r.c.f. for 5 min, and all the supernatant was removed and discarded. The pellet in the extraction mixture was then resuspended in a 100 μl master mix composed of 88 μl of DNA-free water, 10 μl of 10X GREEN+ buffer, 1 μl of prepGEM, and 1 μl of lysozyme. The mixture was vortexed briefly for about 3 s and incubated at 37°C for 15 min, 75°C for 10 min, and 95°C for 5 min. The lysate was centrifuged for 1 min to pellet cell debris, and the supernatant containing the isolated genomic DNA was transferred to a fresh tube and held at +4°C for further downstream analyses.

PCR amplification involved amplifying the 16S rRNA gene from the lysates, using two primers widely applied for identification of bacteria (Lane, 1991; Eid et al., 2020). Lysate (1 μl) was added to a reaction mix containing 10 pmol of primer 27F (AGAGTTTGATCMTGGCTCAG) and 10 pmol of primer 1492R (CGGTTACCTTGTTACGACTT), 1X EmeraldAmp® Max HS PCR Master Mix (Takara) and water to a total volume of 20 μl. A negative control, which excluded the template DNA was always included. The PCR was performed in a Biometra T1 Thermocycler (Analytika Jena) with the following program: 95°C for 10 s, 35 cycles of (98°C for 10 s, 55°C for 30 s, and 72°C for 90 s), a final extension at 72°C for 5 min, and a hold at 10°C. PCR products were separated by gel electrophoresis on 1% (w:v) agarose gels in 1X Tris Acetate—EDTA (1XTAE, 40 mM Tris-Acetate, 1 mM EDTA) buffer and 1X SYBR® Safe DNA Gel Stain (Invitrogen) at 100 V for 40 min and photographed using a UVIDOC HD6 machine (UVITEC Cambridge). Phosphate groups were removed from residual dNTPs by adding 0.5 U Shrimp Alkaline Phosphatase (rSAP, New England BioLab) and unincorporated primers removed by adding 2.5 U Exonuclease 1 (Exo, New England BioLab) to the PCR and incubating at 37°C for 30 min followed by an 80°C for 15 min to denature these enzymes.

### Sequencing and phylogenetic analyses

PCR products for the 16S rRNA gene, obtained using 27F-1492R primers were submitted to the Massey Genome Centre, Massey University, Palmerston North for dideoxy sequencing on an ABI 3730 DNA analyzer (Perkin Elmer). Products were sequenced in both directions using 27F and 1492R and internal primer reported in Schwieger and Tebbe (1998) (Com1 CAGCAGCCGCGGTAATAC). ABI electrophoretograms were edited and assembled in Geneious 9.1.8 https://www.geneious.com.

16S rRNA gene sequences from isolates were matched to an EzBioCloud 16S rRNA gene data base https://www.ezbiocloud.net/ using the USEARCH heuristic algorithm. In this database, 16S rRNA genes are obtained from NGS genome assemblies (Edgar, 2010; Yoon et al., 2017). The 16S rRNA gene sequences from isolates were also matched to a 16S rRNA gene SILVA database (v. 1.38; updated 27.08.20; Quast et al., 2013), using the Spaghetti 16S amplicon bioinformatics pipeline https://github.com/adlape95/Spaghetti.

The lysate sequences and their best matches were aligned using MUSCLE https://www.ebi.ac.uk/Tools/msa/muscle/ (Edgar, 2004) to produce a multiple sequence alignment. The data were exported in CLUSTAL format (Thompson et al., 1994). The 5′ and 3′ ends of this alignment were trimmed to remove uncertain terminal regions of the multiple sequence alignment. A text editor https://www.emeditor.com/ was used for this purpose. These data were loaded into SplitsTree5.0 (Huson and Bryant, 2005) and visualized as a Neighbor-Net splits graph (Bryant and Moulton, 2004). The Neighbor-Net algorithm first determines a circular ordering of the pairwise distances between taxa and then uses a least squares criterion to estimate the length of branches in the splits graph (Winkworth et al., 2005). Neighbor-Net will only produce a tree-like graph if the pairwise distances are suitable for reconstructing a phylogenetic tree. Splits graphs are box-like if the data are not a good fit to a bifurcating tree of evolution. The latter can occur due to sequencing errors, stochastic error, recombination due to hybridization or gene conversion, as well as lateral gene transfer (Huson, 1998; Bryant and Moulton, 2004). Having determined the suitability of the sequences for tree building, an unrooted maximum likelihood tree was constructed for the data matrix using PhyML (Guindon et al., 2010; options chosen: GTR + gamma substitution model, four categories, the alpha value was estimated for each bootstrap replicate: 100 replicates; NNI and SPR branch swapping was used for tree searches with each of the 100 bootstrap replicates).

## Results

### Cave isolates

**Figure 2:**
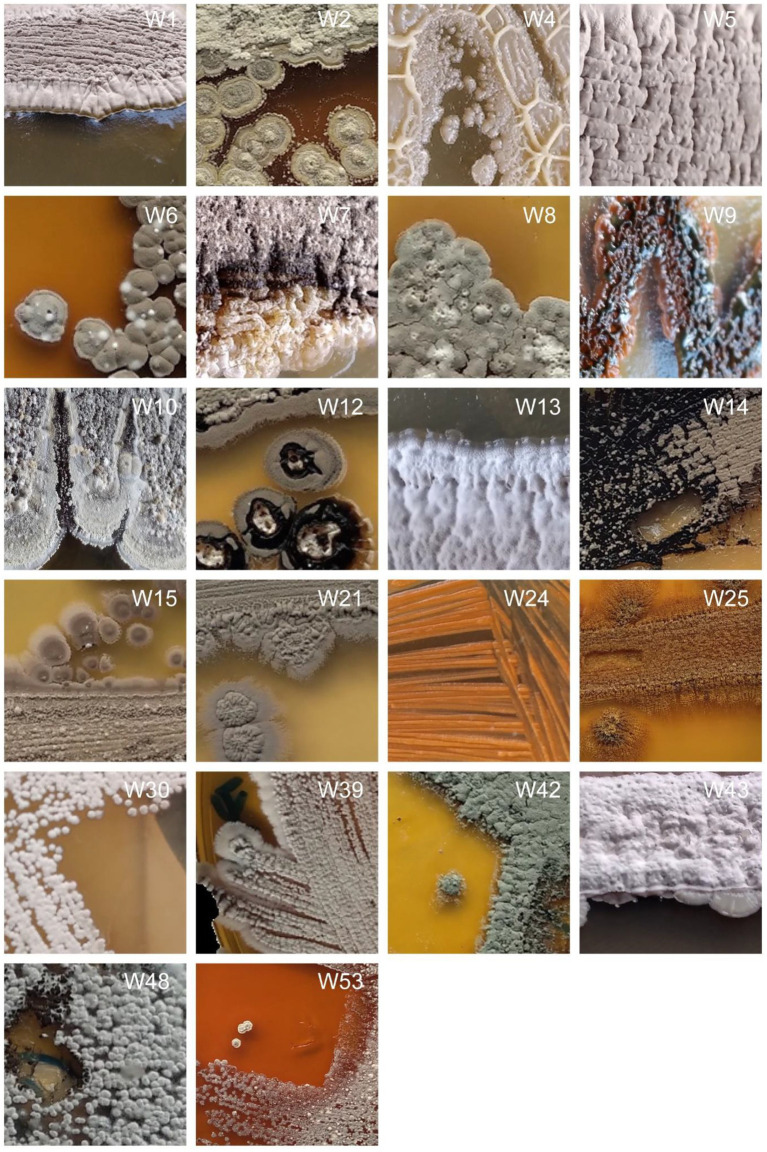
Physical appearances of selected strains isolated from the caves on different isolation media. Photographs are not available for all isolated strains.

**Figure 3:**
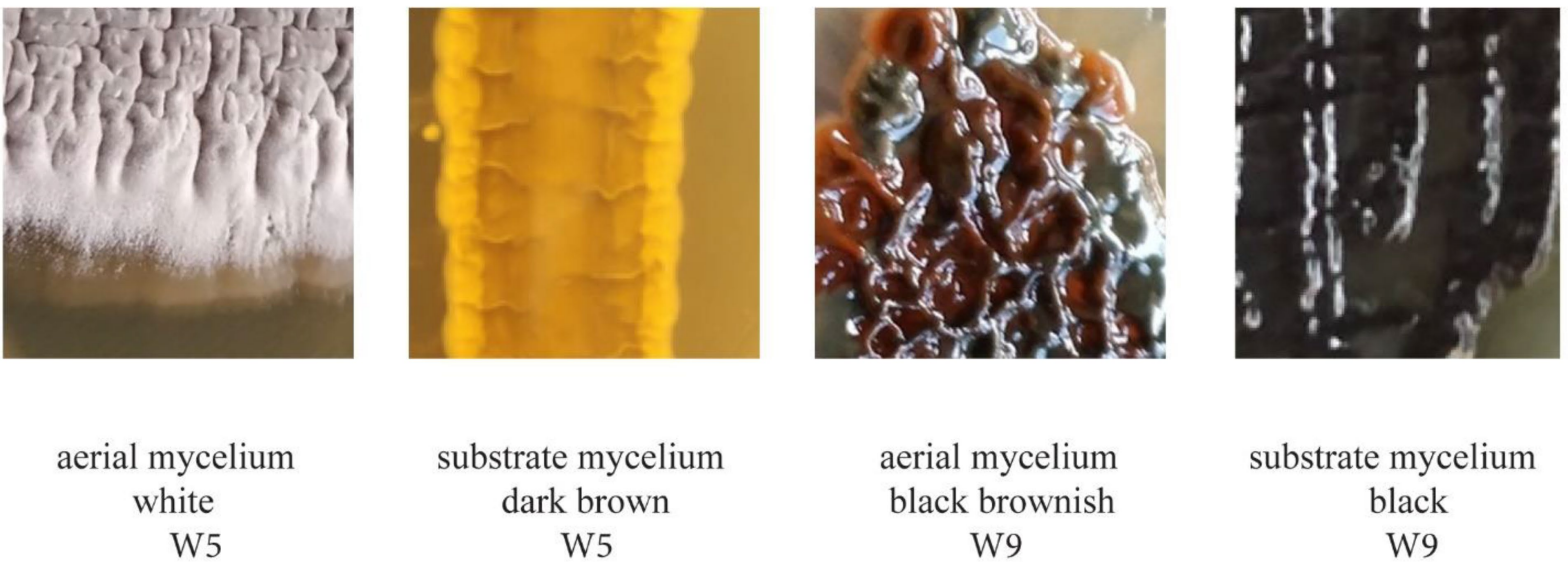
Difference in color between the aerial mycelium and the substrate mycelium of strains W5 and W9.

Cultured bacteria with Actinomyctete-like morphologies for which we have also obtained partial 16S rRNA gene sequences are shown in Figure 2 and Supplementary Table S4. Differences in the color of aerial and substrate mycelium for two isolates are shown in Figure 3. Soil and bat guano produced the highest number of colony forming units (CFUs), with the greatest number being recovered on Humic Vitamin Agar (HVA), and the lowest number of colonies recovered on Starch Casein Agar (Supplementary Table S3). Most CFUs were recovered with the wet heat pretreatment, while a significantly smaller number of colonies were obtained when no pretreatment was used. Pure cultures for each of the 55 cave isolates were named: NW1 and W2-W55. Most Actinomycetes produce pigments which can cause color change of the isolation medium or color differences between the aerial mycelium and the substrate. Close examination of colony morphology showed that most of the cave isolates produced white spores above their respective aerial mycelium and rough, leathery, or powdery aerial mycelium with growth of substrate mycelium, which are recognized characteristics of the Actinomycetes when cultured (Li et al., 2016).

### Antibacterial activity

Of the 55 cave isolates studied, 34 showed medium to large zones of growth inhibition in the cross-streak test measured against at least one of the five test organisms: *Staphylococcus aureus, Streptococcus aureus, Enterococcus faecalis, Klebsiella pneumoniae*, and *Bacillus cereus*. The size of zones of inhibition against the five test microorganisms is indicated in Table 1 and illustrated for two isolates in Figure 1. Overall, for each pathogen tested, 50% of the cave isolates showed inhibitory activity *against Staphylococcus aureus,* 47% against *Streptococcus aureus*, 50% against *Enterococcus faecalis*, 32% against *Klebsiella pneumoniae*, and 41% against *Bacillus cereus* (Table 1). Of the 34 bioactive isolates, two (W14 and W43) produced large zones of inhibition. W14 produced a zone of inhibition of 37 mm against all five bacteria tested and W43 produced a zone of inhibition of 39 mm for four pathogens excluding *Bacillus cereus* (Table 1; Figure 1). In summary, Table 1 provides an indication of potential bioactivity in 34 of our isolates.

**Table 1:** Screening of cave isolates using cross streak method against five pathogenic bacteria.

**Cave Isolates**	**Pathogenic bacteria and average zone of inhibition (in mm)**				
	***Staphylococcus aureus***	***Streptococcus aureus***	***Enterococcus faecalis***	***Klebsiella pneumoniae***	***Bacillus cereus***
NW1	8	0	16	0	0
W2	8	8.2	7	0	10
W3	6	0	12	0	0
W4	0	7.5	0	6.5	3
W5	0	12	0	8	6
W6	0	9.2	0	0	0
W7	0	5.6	0	0	0
W8	0	0	0	14	18.5
W9	19	17	14	16	15.2
W10	0	4.2	8	0	0
W11	14	20	25	28	37
W12	9.1	8	0	0	0
W13	18	0	14	0	35
W14	37	37	37	37	37
W15	0	10	0	13	0
W16	0	5.4	0	0	0
W20	7.4	3.8	0	2	0
W21	0	0	14	0	11
W22	7	0	39	0	0
W24	12.5	8	0	14	5
W25	0	8	11	10	0
W27	15	15	15	0	0
W30	0	0	10.2	0	0
W38	0	5	0	2	0
W39	0	0	6.5	0	6.5
W42	8.6	0	8.2	5.2	6
W43	39	39	39	39	2
W44	0	7.2	4.2	0	0
W47	12.5	10	5.3	0	0
W48	3.5	0	14	0	16
W51	5.2	6.5	0	0	0
W52	7.5	0	5.2	0	12
W53	2	0	30	30	30
W54	3.5	3.2	2	0	3

Inhibition zone are given in mm. Criteria for scoring followed the protocol by Rahman et al. (2011) Biotechnol. Res. Int Article ID 857925. doi:10.4061/2011/857925.

### Taxonomic assignment of cave isolates based on 16S rRNA analyses

**Figure 4:**
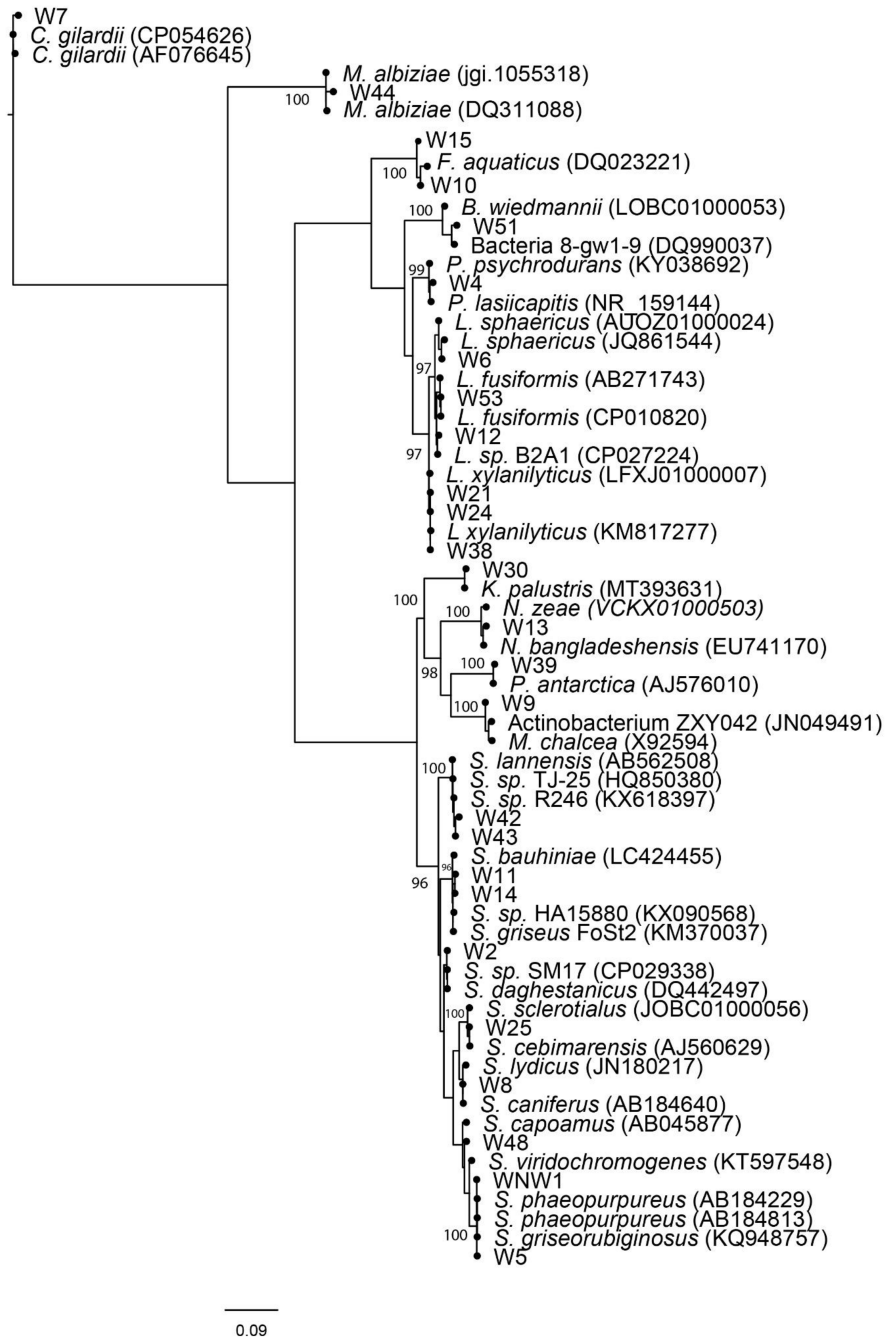
Weighted maximum likelihood tree, arbitrarily rooted on the branch leading to Proteobacteria. Non-parametric bootstrap values >95% have been indicated. Culture (NW1, W2–W54) isolates together with EzBioCloud and SILVA V1.38 database matches have been shown. Relative branch lengths are indicated, and scale length is indicated in substitutions per site.

Pairwise alignment scores for partial 16S rRNA gene sequence are given in Supplementary Table S4. Figure 4 visualizes the phylogenetic relationships between isolates and best matches. The suitability of the data for tree building analyses was first confirmed using Neighbor-Net (Figure not shown). The isolates were identified as belonging to taxa from three different phyla: Actinomycetota, Bacillota, and Pseudomonadota. Most isolates belonged to the genus *Streptomyces* with eight strains (38%), followed by*, Lysinbacillus Fontibacillus and Mesorhizobium* with two strains (9%) each, and *Bacillus, Pseudonocardia, Kocuria, Micromonospora, Nonomuraea, Psychrobacillus,* and *Cupriavidus* shared the same number with one strain each (5%). Actinomycetota (*Streptomyces, Pseudonocardia, Kocuria, Micromonospora*, and *Nonomuraea*) was the most well represented phylum among the sequenced bioactive cultures at 55%, followed by phylum Bacillota (*Bacillus, Lysinbacillus, Fontibacillus*, and *Psychrobacillus*) at 35% and phylum Proteobacteria (*Mesorhizobium* and *Cupriavidus*) at 10%. The best matches differed between the SILVA and EzBioCloud databases but in all cases, matches were made to sequences with greater than 97% similarity. Figure 4 illustrates that the culture conditions recovered a wide genetic diversity of *Streptomyces* species, rare Actinomycete species and *Lysinibacillus* species. Of the two isolates assigned to phylum Pseudomonadota. W44 had greater than 98% sequence similarity with *Mesorhizobium albiziae* and W7 99% sequence similarity to *Cupriavidius gilardii*.

The morphology of cultured strains was compared to the morphology of their closest database matches where information on morphology was available. Images of the closest match, or representative strains from the same genus, showed a close resemblance with isolates W4, W5, W8, W9, W39, W43, and W51 on the culture media used for their isolation (Supplementary Table S4).

### Bioactivity of closest relatives of cave isolates

Figure 4 shows that the 16S rRNA sequences are closely related to bacteria with a diverse range of bioactivities. This includes isolates most closely matching: bacterial reference strains with antimicrobial properties (NW1, W2, W4, W5, W8, W9, W11, W12, W14, W25, W30, W42, and W48), bacteria useful for bioremediation of toxic wastes or biomining (W4, W10, W12, W15, W39, W51, and W53), bacteria that produce bioplastics (W51) bacteria that produce pesticides (W6, W21, W24, and W38), bacteria that have anticancer properties (W8, W13, and W25), bacteria that promote plant growth (W4, W9, W21, W24, and W44), and bacteria used in cosmetics (W30).

## Discussion

The pre-treatments and selective culturing conditions employed in the present study recovered a wide diversity of strains exhibiting antibacterial properties. Most bioactive bacteria were identified as genetically diverse Actinomycetes. High diversity of Actinomycetes has also been reported from other cave systems outside of Oceania, and our study adds to the growing appreciation that caves, particularly cave floors and speleothems, harbor a wide range of Actinomycetes and secondary metabolites (Nimaichand et al., 2015; Axenov-Gribanov et al., 2016a; Maciejewska et al., 2018; Hamedi et al., 2019; Long et al., 2019; Rangseekaew and Pathom-aree, 2019; Wiseschart et al., 2019; Prescott et al., 2022).

The high genetic diversity of bacteria with a potentially diverse range of bioactivities isolated is perhaps not surprising. Oceanic caves are oligotrophic and tropical, providing strong evolutionary selection pressures for microbes with secondary metabolisms needed for their growth and defense (Cuezva et al., 2009; Hamedi et al., 2019). The physiochemical properties of the cave that was sampled shares similarities with previously studied caves outside of Oceania. These include absence of light (zero Lux; Ghosh et al., 2016), little energy exchange with their external environments (Kambesis, 2007), and many formations of secondary mineral deposits known as speleothems (stalactite and moonmilk in Supplementary Figure S2; Bontemps et al., 2021). However, cave type, local geology, and surrounding vegetation can all create heterogeneity of mineral and organic surfaces and this can impact cave microhabitats (Ghosh et al., 2016, 2020; Prescott et al., 2022). Specific factors such as pH, temperature, salinity, and resource availability on rock surfaces (e.g., oligotrophic conditions on rock surfaces and limited availability of organic carbon), temperature and humidity can differ within and between caves determine opportunities for microbial growth (Cuezva et al., 2009; Bontemps et al., 2021; Iquebal et al., 2021). Measurements of pH, temperature, and salinity in the Fiji cave differ from measurements reported for other studied caves. The extent to which these differences might have shaped microbial genomes in the cave is yet undetermined. Many of the bacteria cultured have high levels of similarity in their 16S rRNA sequences to previously isolated bacteria, perhaps suggesting that previously discovered species have been isolated (Kim et al., 2014). This might be the case. However, previous genomic and metabolite studies have also shown that taxa with highly similar or even identical 16S rRNA sequences can also have very different secondary metabolite profiles (Sekurova et al., 2019). The presence and expression of biosynthetic gene clusters (BGCs) is known to be influenced by abiotic and biotic stresses in natural environments (Undabarrena et al., 2021), and conditions in the Fiji cave may have selected for novel biosynthetic gene clusters (Van der Meij et al., 2017).

The stalactite, moon milk, bat guano, and cave soil samples in our study varied in their biological and physical nature. The cave soil and moonmilk samples were nearly neutral in pH, while the bat guano had slightly acidic pH, possibly due to mineralization of animal inputs resulting in the production of nitric acid and sulfuric acid (Long et al., 2019). A pH of 7.2 was used in selective media when culturing samples. Salinity was also a consideration (Supplementary Table S1). The rationale behind mimicking natural environments in the culture conditions was to create environments in which the bacteria would continue to produce secondary metabolites (Trenozhnikova and Azizan, 2018). The biosynthesis of secondary metabolites is known to be very dependent on internal and external signals (Pang, 2018). Furthermore, Actinomycetes are known to be slow growing and culture conditions need to promote their growth and to inhibit the growth of competing fast growing organisms (Tiwari and Gupta, 2012). Our combination of different selective media supplemented with chemicals inhibitors as well as pretreatments was successful with 34 of the 55 (62%) isolates with Actinomycete-like morphology expressing antimicrobial activity against at least one pathogenic strain of bacteria under the test conditions. The CFU results showed that following pretreatments and growth on selective media, the bacterial diversity was greatest in bat guano and lowest in moonmilk samples (bat guano > soil > stalactite > moonmilk). Similar relative diversities have been reported in other cave studies (Hamedi et al., 2019; Long et al., 2019). In our study, HVA medium produced the highest diversity of bacteria with all pretreated samples followed by AIA, SCA, and ISP5. Previously, HVA has been shown to support the growth of diverse Actinomycetes by activating the germination of their spores (Subramani and Aalbersberg, 2013). SCA and AIA have also been found to favor growth of Actinomycetes when supplemented with inhibitors of fungi and other fast-growing bacteria (Rashad et al., 2015; Almalki, 2020). Most Actinomycetes in the present study were recovered from the cave soil, a finding also consistent with previous cave studies (Long et al., 2019). Zones of inhibition between studies are not strictly comparable as size will be affected both by antibiotic effect and also rate of diffusion. However, it is perhaps notable that both W14 and W43 produced large zones of inhibition (37 and 39 mm respectively) against pathogenic bacteria more than 2x the size of the inhibition zones produced by the other bioactive bacteria inhibition zones isolated. The zone of inhibition reported by Yücel and Yamac (2010) for *Streptomyces* sp. 1,492 recovered from a Turkish karstic cave was 15 mm in tests against Methicillin-resistant *Staphylococcus aureus* (MRSA), while the most bioactive Actinomycetes isolated from the Shuanghe karst cave in Asia S142 (*Streptomyces badius*) and S761 (*Actinoplanes friuliensis*) produced zones of inhibition 17.9 and 15.2 mm in size, respectively, (Long et al., 2019). The activities of W14 and W43 are certainly worthy of further investigation. However, it is also worth noting that cave dwelling Actinomycetes previously reported with small inhibition zones have also been found to produce unique bioactive compounds. For example this includes hypogeamicines A–D, produced by *Nonomuraea specus* (Derewacz et al., 2014) and Xiakemycin A, a new pyranonaphthoquinone (PNQ) antibiotic produced by *Streptomyces* sp. CC8-201 (Jiang et al., 2015). Thus, size of inhibition zones alone is insufficient as a criterion to select isolates for further study.

*Streptomyces* represent the largest genus of Actinomycetes and constitute the majority of cultured Actinomycetes (Hamedi et al., 2019; Long et al., 2019; Sripreechasak and Athipornchai, 2019). Two-thirds of known antibiotics are produced by *Streptomyces* species (Fernández-Martínez, 2019). They are ubiquitous in terrestrial and marine environments with the greatest diversity recorded from terrestrial habitats (Quinn et al., 2020). Whatever the habitats, the natural products produced by *Streptomyces* under normal and extreme conditions exhibit great structural diversity and great biological activity (Procopio et al., 2012; Giordano, 2020). Rediscovery of known secondary metabolites from *Streptomyces* species has redirected scientists to the discovery of rare Actinomycetes with claims that *Streptomyces* species offer no significant potential biological resource for new antibiotics (Subramani and Sipkema, 2019). The reason for the high recovery of *Streptomyces* in caves may lie in their dispersal of spores, the ability to utilize a wide variety of nutrient sources in synthetic rich media, and their faster growth compared to other genera, recognized as rare Actinobacteria (Subramani and Aalbersberg, 2013; Maciejewska et al., 2016). While repeated rediscovery of similar metabolites in *Streptomyces* species has turned focus onto rare (non *Streptomyces*) Actinomycetes (Sekurova et al., 2019), the extent of the genetic diversity of bioactive *Streptomyces* species, also evident in our phylogenetic analyses, suggests it might be short sighted to cease the search for new species of *Streptomyces* and novel compounds.

W9 and W13, W30 and W39 were found to be rare Actinomycetes and closely related to *Micromonospora chalcea*, *Nonomuraea zeae*, and *Nonomuraea bangladeshensis*, *Kocuria palustris*, and *Pseudonocardia antarctica*, respectively. *Micromonospora*, *Nonomuraea*, *Pseudonocardia*, and *Kocuria* have all previously been reported from caves (Belyagoubi et al., 2018; Hamedi et al., 2019; Long et al., 2019). They were isolated here from cave bat guano and cave soil (Supplementary Table S4). Bat guano has been shown to be an excellent source of rare Actinomycetes (Long et al., 2019). Genera such as *Micromonospora, Nocardia, Pseudonocardia*, *and Kocuria* closely related to isolates recovered in this study have been found to produce chemically unique antibiotics featuring potent activities, such as abyssomicins and proximicins (Hug et al., 2018). W9 produced the greatest streak test inhibition (Table 1) among the rare Actinomycetes recovered and this isolate was assigned to genus *Micromonospora*. The genus *Micromonospora* has been investigated extensively and more than 100 antibiotics have been isolated from diverse *Micromonospora* strains (Boumehira et al., 2016). Hifnawy et al. (2020) discuss the potential of genus *Micromonospora* as a model system for natural product research and report on the discovery of chemical structures, their biological activities and biosynthetic studies in this genus (Hifnawy et al., 2020). Similarly*, Nonomuraea, Pseudonocardia*, and *Kocuria* genus are significant producers of bioactive compounds. *Nonomuraea apecus,* isolated from a Tennessee cave, was found to produce novel bioactive compounds, monomeric hypogeamicins A-D that showed antibiotic activity. A dimeric form of hypogeamicin A, showed cytotoxicity to colon cancer cell line TCT-1 (Derewacz et al., 2014). Riahi et al. (2022) have recently reviewed current knowledge regarding the biology of *Pseudonocardia* species, the great diversity of bioactive natural products produced from *Pseudonocardia* species and the different applications of these products in biotechnology (Riahi et al., 2022).

## Conclusion

An exciting direction for natural products research is the underexplored environments of caves, which host a diversity of Actinomycetes and other bioactive bacteria. To our knowledge, our study is the first to report microbial communities from a natural cave ecosystem of Fiji, and the first to culture bioactive bacteria from Oceania. Pretreatments and selective media were used to isolate a wide genetic diversity of bacteria including *Streptomyces* and rare Actinomycetes, whose frequency of isolation has typically been low in many studies (Subramani and Aalbersberg, 2013; Subramani and Sipkema, 2019). Over 60% of our cultured isolates exhibited antibiotic potential against at least one of the tested pathogenic bacteria. Our study highlights and corroborates the recent report of great microbial diversity in the Oceanic Island caves of Hawaii (Prescott et al., 2022). Our findings are encouraging that many of the bacteria in Oceanic Island caves might be culturable.

## Data availability statement

The 16S rRNA gene sequences presented in our study are deposited in GenBANK, accession numbers OP854818- OP854843.

## Author contributions

AP, RS, and PL: conceptualization. AP carried out culture-based experiments. AP and DK designed and carried out the field work. PL, PM, and AP carried DNA sequence analyses. PL and KC supervised the experiments. AP, PL, PM, DK, KC, and SP contributed to the article and approved the submitted version.

## Funding

The University of the South Pacific provided MSc research funding support to AP for culture-based studies on Oceanic Island cave dwelling Actinobacteria. The Government of Vanuatu provided financial support to support the studies of the lead author AP Massey University and The New Zealand Royal Society Catalyst Fund provided funding to PL to support this research collaboration. Financial support for the project was provided by the University of the South Pacific, Massey University, the New Zealand Catalyst Fund (MAU1707) and the Vanuatu Ministry of Education.

## Conflict of interest

The authors declare that the research was conducted in the absence of any commercial or financial relationships that could be construed as a potential conflict of interest.

## Publisher’s note

All claims expressed in this article are solely those of the authors and do not necessarily represent those of their affiliated organizations, or those of the publisher, the editors and the reviewers. Any product that may be evaluated in this article, or claim that may be made by its manufacturer, is not guaranteed or endorsed by the publisher.

## Footnotes

^1^ http://www.bacterio.net/-classifphyla.html#Actinobacteria
