# Supplementary material for: Isolation, antibacterial screening, and identification of bioactive cave dwelling bacteria in Fiji
Source: Front Microbiol. 2022 Dec 20;13:1012867. doi: 10.3389/fmicb.2022.1012867 (PMC9807670; doi:10.3389/fmicb.2022.1012867)
Supplement: Supplementary file 1 [file Data_Sheet_1.pdf]

Supplementary Materials

Supplementary Table S1 Physiochemical parameters of Fiji cave samples

	soil	stalactite	moonmilk speleothems	bat guano
temperature °C	23.5	25	25	24.2
relative humidity %	70.5	70.5	70.5	70.5
light (lux)	0	0	0	0
pH	7.45	7	7	4.98
salinity ‰	3.8	3	3	4

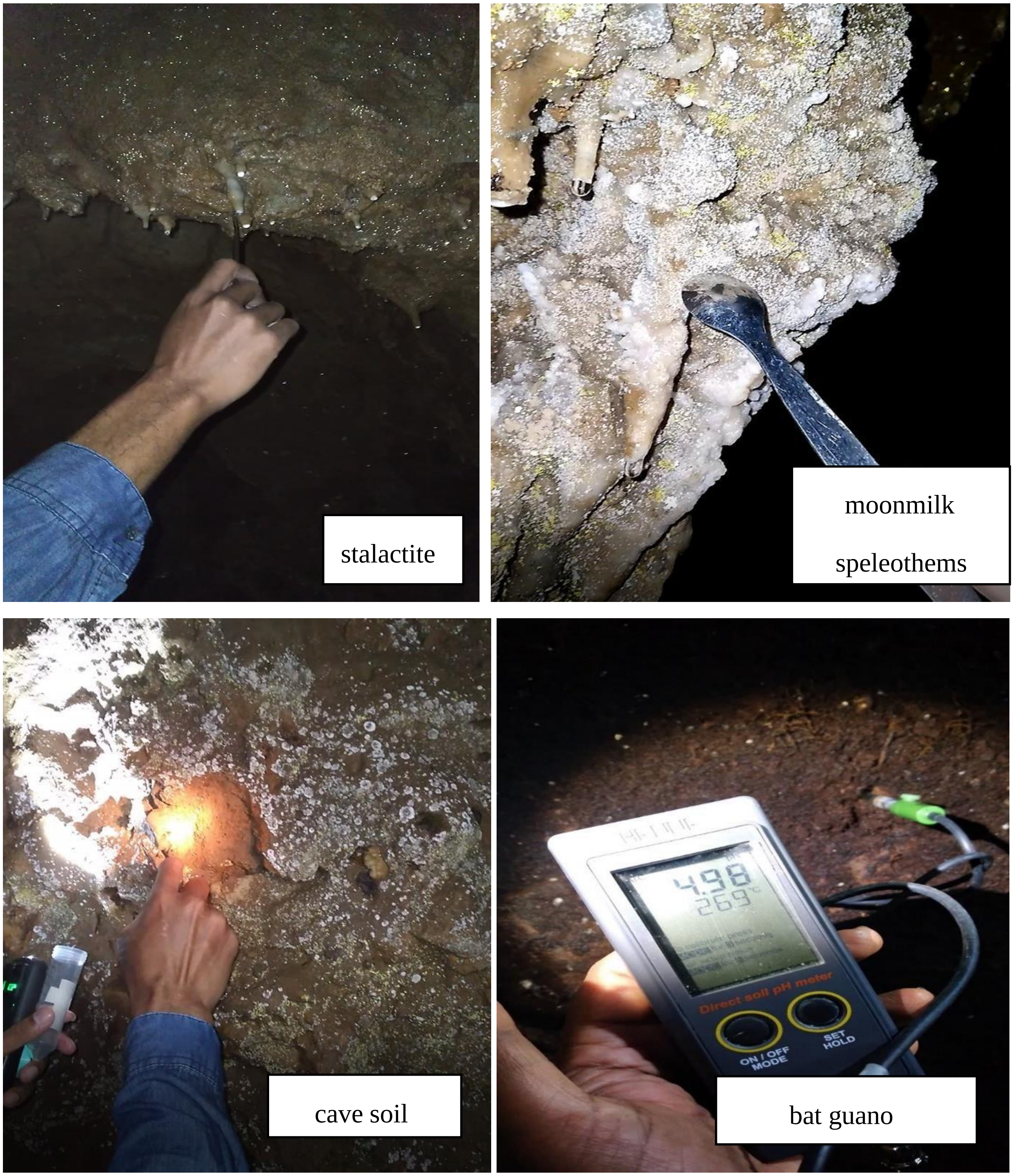

Supplementary Figure S1: Samples collected

Supplementary Table S2 Pretreatments and selective media used in this study

pretreatments	phenol	1 g of environmental sample was added to 1.5% phenol (w/v) with 10 mL of sterile water. The resulting mixture was vortexed for 5 minutes and allowed to settle for 5 minutes. The supernatant was serially diluted with sterile water from 10-1 to 10-4. 100 µL from each dilution was used for inoculating agar plates.
	wet heat	1 g of environmental sample was added to 10 mL of sterile water. The resulting mixture was heated to 60 ºC for 15 minutes using a water bath and then allowed to settle for 5 minutes. The supernatant was serially diluted with sterile water from 10-1 to 10-4. 100 µL from each dilution was used for inoculating agar plates.
selective media	Growth on Humic Vitamin Agar (HVA)	Medium consists of - Humic acid – 1.0 g (Dissolved in 10 mL of 0.2 N NaOH), Na2HPO4 - 0.5 g , KCl – 1.71 g, MgSO4.7H2O – 0.05 g, FeSO4.7H2O – 0.01 g, CaCO3 – 0.02 g, B-Vitamins – 0.5 mg each of thiamine-HCl, riboflavin, niacin, pyridoxine-HCL- inositol, ca-pantothenate, p-amino benzoic acid, and 0.25 mg of biotin. B-vitamins and cycloheximide were filter sterilized by membrane filtration and added to the autoclaved media. pH was adjusted to pH 7.2.
	Growth on Starch Casein agar (SCA)	Glycerol (or starch) – 10.0 g, Casein (Difco-vitamin-free) – 0.3 g, KNO3 – 2.0 g, NaCl – 2.0 g, K2HPO4 – 2.0 g, MgSO4.7H2O – 0.05 g, CaCO3 – 0.02 g , FeSO4.7H2O – 0.01 g, Bacto Agar – 18.0 g, Distilled water – 1,000 mL. pH was adjusted to pH 7.2.
	Growth on Actinomycetes isolation agar (AIA)	Sodium caseinate 2.0 g, L-Asparagine 0.1 g, Sodium propionate 4.0 g, Dipotassium phosphate 0.5, Magnesium sulphate 0.1 g, Ferrous sulphate 0.001 g, Agar 15.0 g. Distilled water – 1,000 mL. pH was adjusted to pH 7.2.
	growth on International Streptomyces Project 5 Agar (ISP5)	L-asparagine (anhydrous basis) – 1.0 g, Glycerol -10.0 g, K_2HPO_4 (anhydrous basis) – 1.0 g, Distilled water – 1.0 Liter, Trace salt Solution – 1.0 mL (The pH of this solution is about 7.0 – 7.4. Should not be adjusted if is within this range, Agar – 20.0 g (Liquefy agar by steaming at 100 ℃ for 15 – 20 minutes). pH was adjusted to pH 7.2.

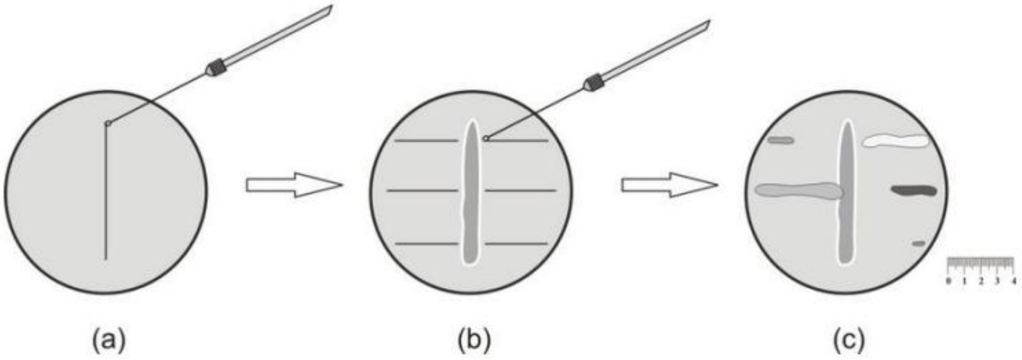

Illustration of cross streak method: from Vasabhai, R-D. (2019). Isolation & characterization of Actinomycetes producing antimicrobial compounds surrounding the soil of different medicinal plants in Saurashtra region of Gujarat: Comparative studies of Indian medicinal plants. Doctoral Thesis, Rk university, Rajkot, Gujarat, India.

Supplementary Figure S2 Cartoon illustrating cross streak method

Supplementary Table S3: The average numbers of CFUs recorded for highest bacterial diversity on selective media used in this study. * Too many to count (TMTC)

medium	sample source	Colony Forming Unit (CFU). (average value of triplicates) per gram
Actinomycetes Isolation Agar (AIA)	stalactite	2.00*10^8
	soil	8.00*10^8
	moonmilk	2.00*10^8
	bat guano	TMTC*
International Streptomyces Agar 5 (ISP5)	stalactite	5.00*10^7
	soil	8.00*10^8
	moonmik	8.00*10^7
	bat guano	9.00*10^8
Starch Casein Agar (SCA)	stalactite	2.00*10^8
	soil	9.00*10^8
	moonmik	2.00*10^8
	bat guano	TMTC
Humic Vitamin Agar (HVA)	stalactite	5.00*10^8
	soil	TMTC
	moonmik	4.00*10^8
	bat guano	TMTC

Supplementary Table S4. Percent Identity of culture 16S rDNA, closest GenBank match and Bioactivity of the closest match

Cave isolate source	% Identity in best match alignments<br><br>i) USEARCH alignments (EzBioCloud database)<br>ii) BLAST score for best MiniMap2 assignments made using Spaghetti (Silva ver 1.38 database)		Colony morphology of cave isolates	Colony morphology of the closest match	Bioactivity of best database match made using two criteria
WNW1<br><br>soil	*Streptomyces griseorubiginosus* DSM 40469<br><br>*Streptomyces phaeopurpureus* (AB184813)	99.65<br><br>99.79	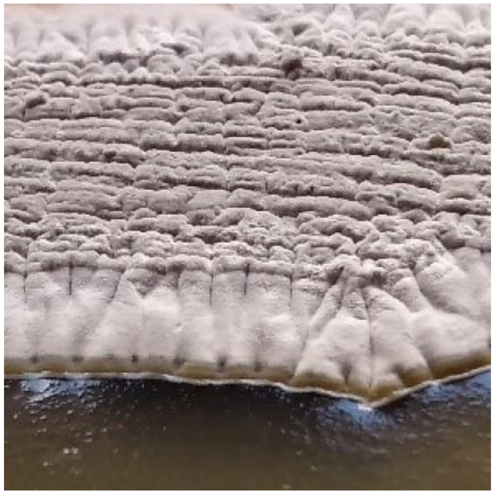	Unpublished (NA)	*Streptomyces griseorubiginosus* has been investigated as a biocontrol agent for the management of cucumber anthracnose (Chai et al., 2022) *Streptomyces phaeopurpureus* is known to produce antifungal proteases and has been investigated as a biofungicide (Palaniyandi *et al*., 2013).

W2<br><br>bat guano	*Streptomyces daghestanicus* NRRL B-5418<br><br>*Streptomyces sp.* SM17 chromosome, complete genome (CP029338)	100<br><br>100	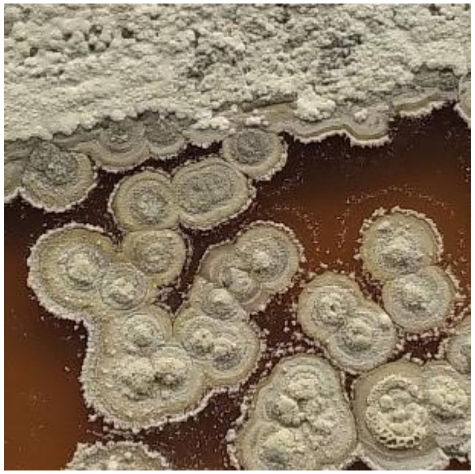		*Streptomyces daghestanicus* is reported to exhibit antimicrobial activity (Tedsree *et al*., 2022).<br><br>*Streptomyces* sp. SM17 possesses antimicrobial activity against Gram-negative and Gram-positive bacteria – including methicillin-resistant *S. aureus* (MRSA), and yeasts (Almeida *et al*., 2019).
W4<br><br>bat guano	*Psychrobacillus lasiicapitis* NEAU-3TGS17<br><br>*Psychrobacillus psychrodurans* strain FJAT-46038 (KY038692)	99.24<br><br>98.81	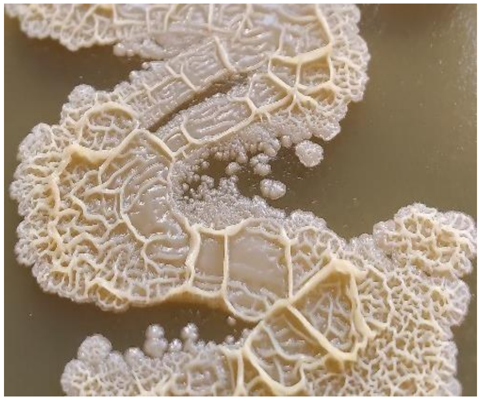	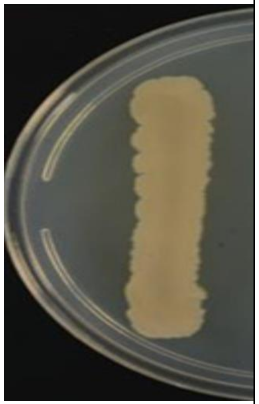	*Psychrobacillus psychrodurans* is a plant growth promoting agent (Xu *et al*., 2018).<br><br>*Psychrobacillus* species are involved in bioremediation (Jeong & Kim, 2015) and as antimicrobial agents (Das *et al*., 2017).

W5<br><br>soil	*Streptomyces griseorubiginosus* DSM 40469<br><br>*Streptomyces phaeopurpureus* NBRC 12899 (AB184229)	99.65<br><br>99.85	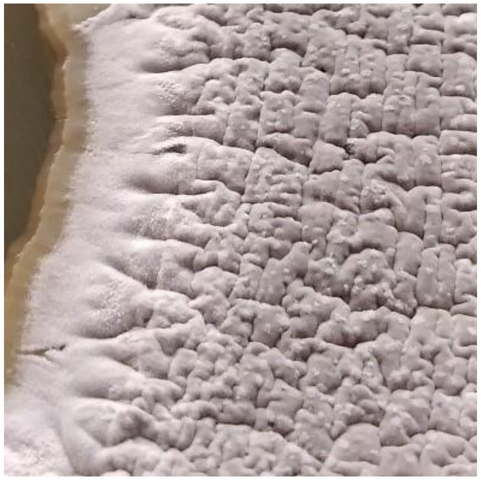	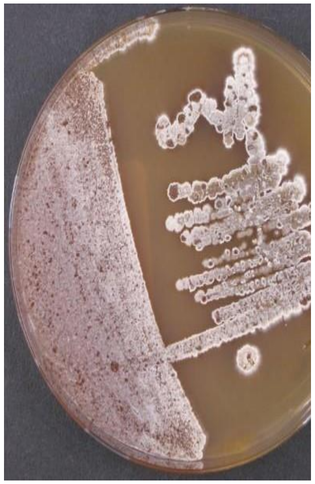<br><br>https://bacdive.dsmz.de/strain/15469	*Streptomyces griseorubiginosus* has been investigated as a biocontrol agent for the management of cucumber anthracnose (Chai *et al*., 2022).<br><br>*Streptomyces phaeopurpureus* is known to produce antifungal proteases and has been investigated as a biofungicide (Palaniyandi *et al*., 2013).

W6<br><br>soil	*Lysinibacillus sphaericus* KCTC 3346<br><br>*Lysinibacillus sphaericus* strain Pp10 (JQ861544)	99.51<br><br>99.27	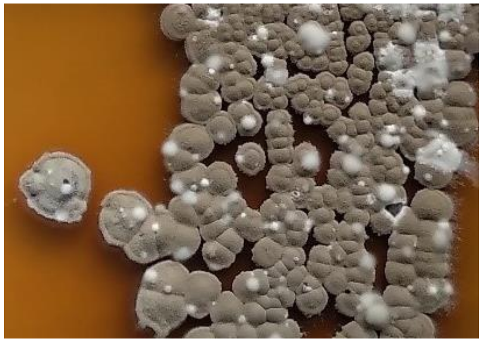		*Lysinibacillus sphaericus* has been investigated for its potential used in for bioremediation and biomining, especially related to gold extraction (Bustos et al., 2018).<br>It is an entomopathogen, and widely used to produce the active ingredient in mosquito insecticides (Cavados *et al*., 2017).
W7<br><br>soil	*Cupriavidus gilardii*<br><br>LMG 5886	99.38	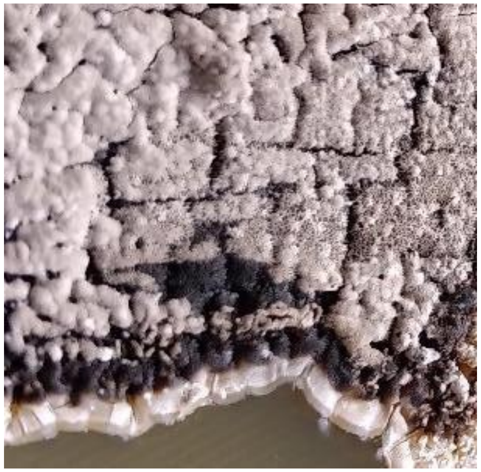		*Cupriavidus gilardii* has been investigated for bioremediation of soils contaminated with the herbicide 2-Methyl-4-chlorophenoxyacetic acid (MCPA) with positive outcomes (Pan *et al*., 2022).

W8<br><br>soil	*Streptomyces caniferus* NBRC 15389<br><br>*Streptomyces lydicus* strain S3521 (JN180217)	99.79<br><br>99.37	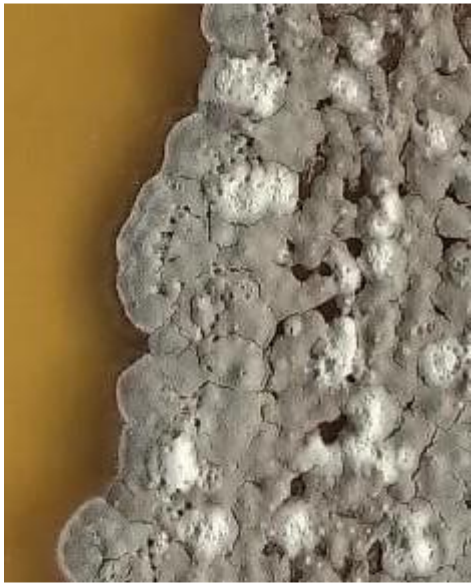	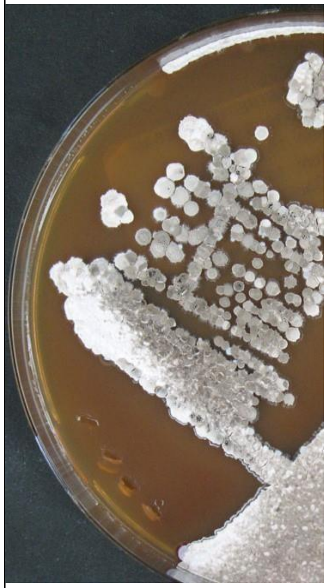<br><br>https://bacdive.dsmz.de/strain/15474	*Streptomyces caniferus* harbor bioactive compounds including antitumor macrolides (Pérez *et al*., 2016).<br><br>*Streptomyces lydicus* has various antimicrobial activities (Lertcanawanichakul *et al*., 2015).

W9<br><br>bat guano	*Micromonospora chalcea* DSM 43026<br><br>Actinobacterium ZXY042 (JN049491)	99.57<br><br>99.0	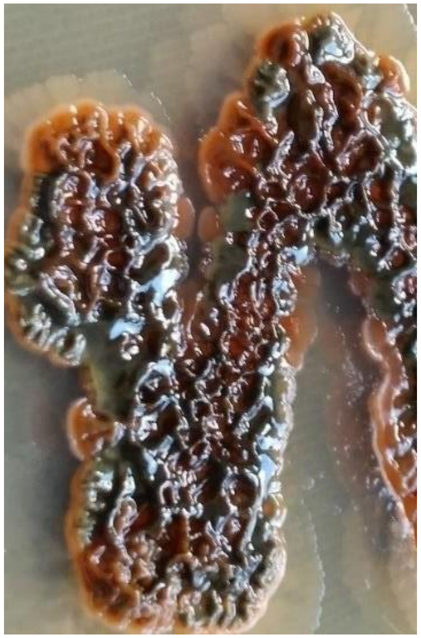	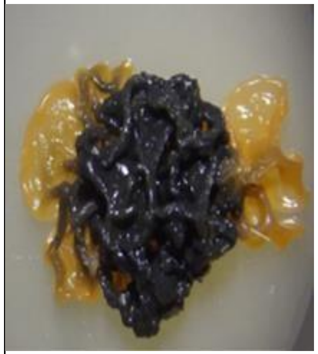<br><br>https://www.frontiersin.org/articles/10.3389/fmicb.2015.01341/full	*Micromonospora chalcea* has seven 16-membered macrolide antibiotics structurally close to rosamicin with antibacterial activity against Gram-positive bacteria (Hifnawy *et al.*, 2020).<br><br>*Micromonospora chalcea* is a potential plant biostimulant. It was reported to enhance the growth of *Salicornia bigelovii* (El-Tarabily *et al*., 2019).
W10<br><br>bat guano	*Fontibacillus aquaticus* GPTSA19<br><br>*Fontibacillus aquaticus* GPTSA19	99.31<br><br>99.24	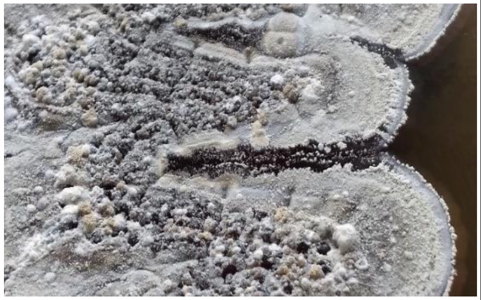		*Fontibacillus aquaticus* is reported to reduce graphite oxide contaminants (Chouhan *et al.*, 2016).

W11<br><br>soil	*Streptomyces bauhiniae* Bv016<br><br>*Streptomyces griseus* strain FoSt2 (KM370037)	99.65<br><br>99.50	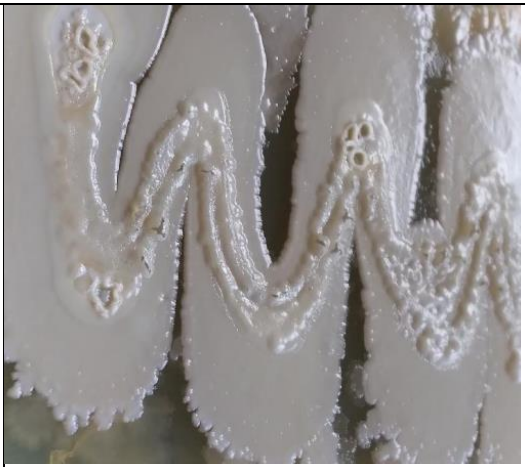		*Streptomyces griseus* is well known for the production of streptomycin, which is the first aminoglycoside antibiotic, discovered more than 60 years ago. This species has since been found to produce a large number of other bioactive metabolites and the genome sequence of this species has enormous potential in drug discovery (Yasuo *et al*., 2008).<br><br>*Streptomyces* are the richest source of bioactive compounds used for the treatment of various ailments (Donald *et al*., 2022).
W12<br><br>soil	*Lysinibacillus fusiformis* NBRC 15717<br><br>*Lysinibacillus*_sp B2A1 (CP027224)	99.24<br><br>99.04	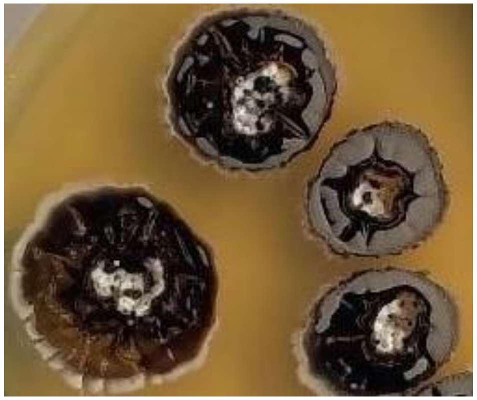		*Lysinibacillus fusiformis* is proficient for the bioremediation of soluble chromate-contaminated soil by reducing its toxicity and bioavailability (Huang *et al*., 2016). It has greater antagonistic activity against antibiotic-resistant pathogens (*Pseudomonas aerugninosa*, *Klebsiella* sp., and *Streptococcus pneumonia*) (Abideen & Babuselvam, 2014). It is used in the treatment of wastewater rich in metal ions (Taieb *et al*., 2021).

W13<br><br>soil	*Nonomuraea zeae* DSM 100528<br><br>*Nonomuraea bangladeshensis* strain 13651O (EU741172)	99.08<br><br>99.72	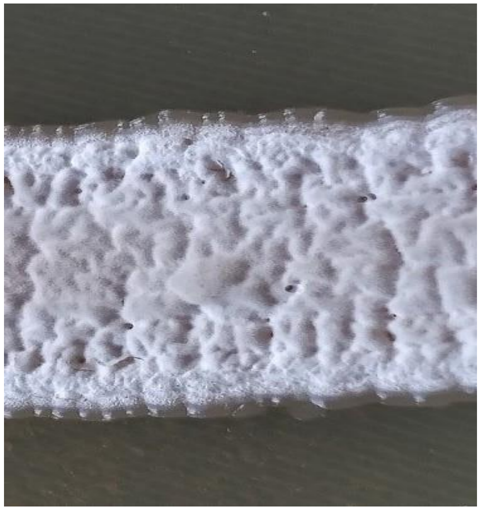		*Nonomuraea* species are reported to have anticancer and antibacterial activity (Nakaew *et al*., 2009).

W14<br><br>soil	*Streptomyces bauhiniae* Bv016<br><br>*Streptomyces* sp. strain HA15880 (KX090568)	99.58<br><br>99.30	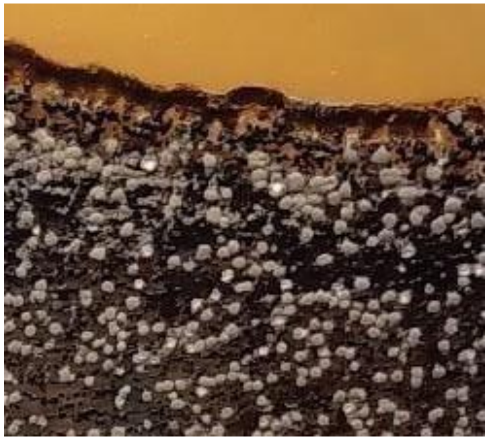		*Streptomyces* are the richest source of bioactive compounds used for the treatment of various ailments (Donald *et al*., 2022).

W15<br><br>soil	Fontibacillus aquaticus GPTSA19<br><br>*Fontibacillus aquaticus* (DQ023221)	99.31<br><br>99.17	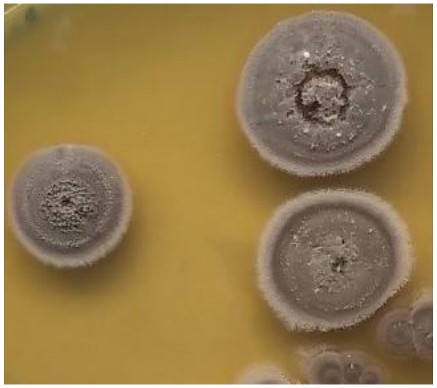		*Fontibacillus aquaticus* is reported to reduce graphite oxide contaminants (Chouhan *et al*., 2016).
W21<br><br>soil	*Lysinibacillus sp.* SR-86<br><br>*Lysinibacillus xylanilyticus* strain IHB B 14504 (KM817277)	100<br><br>99.59	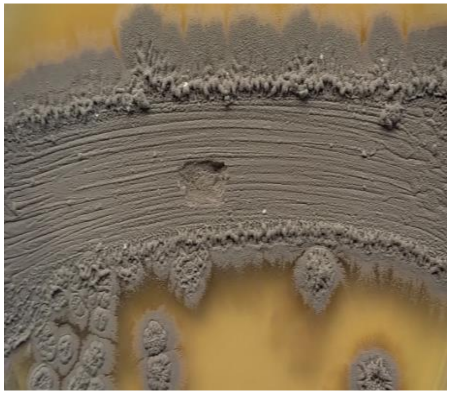		*Lysinibacillus xylanilyticus* has been identified as a potential plant biostimulant (Ahsan et al., 2021). It can be used as an effective biocontrol measure for *Argyrotaenia sphaleropa*, a lepidopteran pest in deciduous fruit orchards (Abreo *et al*., 2018).
W24<br><br>soil	*Lysinibacillus sp.* SR-86<br><br>*Lysinibacillus xylanilyticus* strain IHB B 14504 (KM817277)	100<br><br>99.59	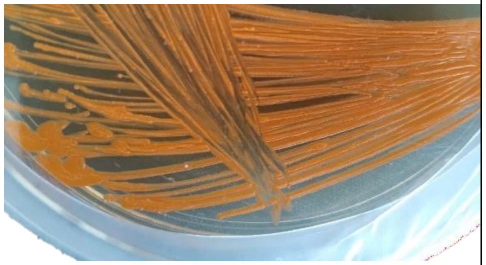		*Lysinibacillus xylanilyticus* is identified as a potential plant biostimulant (Ahsan *et al*., 2021). It can be used as an effective biocontrol measure for *Argyrotaenia sphaleropa*, a lepidopteran pest in deciduous fruit orchards (Abreo *et al*., 2018).

W25<br><br>bat guano	*Streptomyces sclerotialus* NRRL ISP-5269<br><br>*Streptomyces cebimarensis* SS99BA-2 (AJ560629)	99.51<br><br>99.79	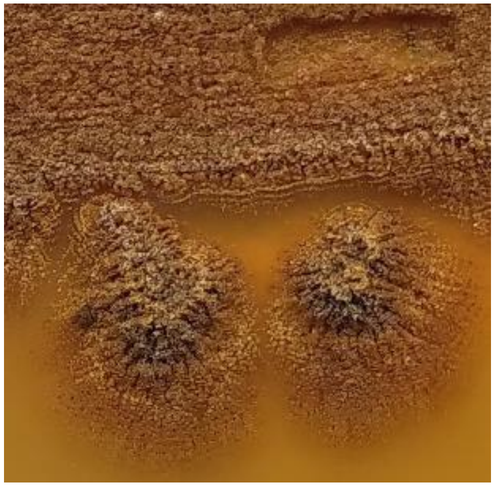		*Streptomyces sclerotialus* has been used to produce silver nanoparticles (AgNPs) for insecticidal and antibacterial purposes (Raguvaran *et al*., 2022).<br><br>*Streptomyces cebimarensis* has cytotoxic and antitumor activities (Romano *et al*., 2014).

W30<br><br>soil	*Kocuria palustris* DSM 11925	99.82	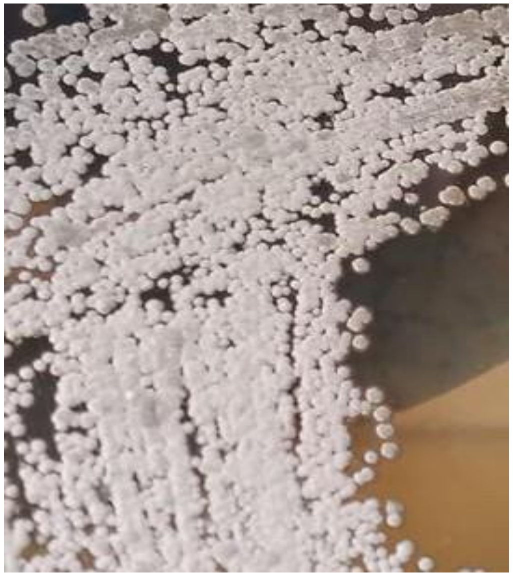		*Kocuria palustris* has fungicidal activity against *Fusarium oxysporum* (Setiawan et al., 2022). It harbors a rare carotenoid with higher antioxidant and photoprotective properties suitable for cosmetic applications (Mendes-Silva *et al*., 2021)

W38<br><br>soil	*Lysinibacillus sp.* SR-86<br><br>Lysinibacillus_xylanilyticus (KM817277)	99.79	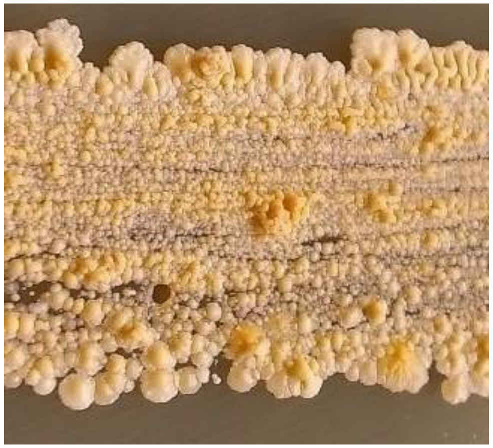		*Lysinibacillus xylanilyticus* has been identified as a potential plant biostimulant (Ahsan et al., 2021). It can be used as an effective biocontrol measure for *Argyrotaenia sphaleropa*, a lepidopteran pest in deciduous fruit orchards (Abreo *et al.*, 2018).
W39<br><br>soil	*Pseudonocardia antarctica* DVS 5a1	99.82	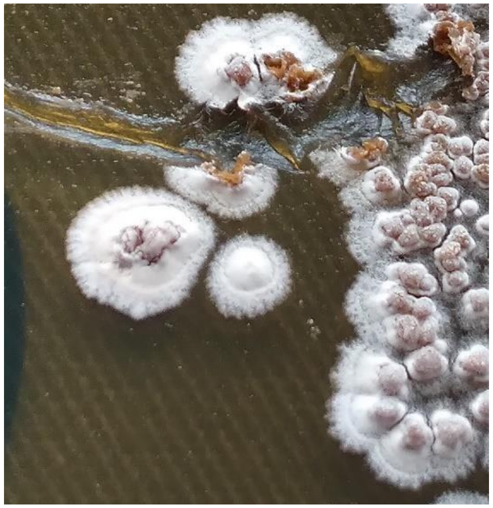	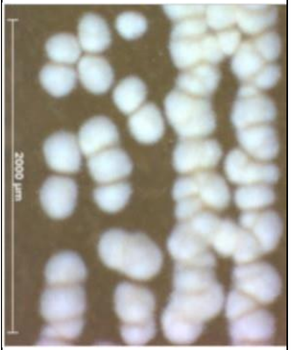<br><br>http://actinobase.org/index.php/File:Ps.png	The genus *Pseudonocardia* has contaminant-degrading properties and an antimicrobial profile (Riahi *et al.*, 2022).

W42	*Streptomyces lannensis* TA4-8<br><br>*Streptomyces sp*. strain R246 (KX618397)	99.28<br><br>97.8			*Streptomyces lannensis* is a source for actinomycin D; a compound responsible for the antagonistic activity against multi-drug resistant pathogens (Dahal *et al.*, 2020).
W43<br><br>soil	*Streptomyces lannensis* TA4-8<br><br>*Streptomyces sp*. TJ-25 (HQ850380)	99.86	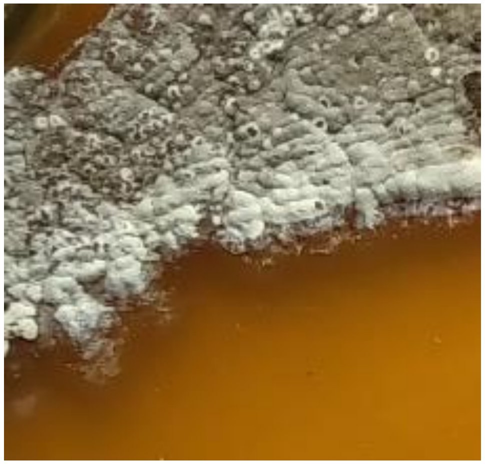	NA<br>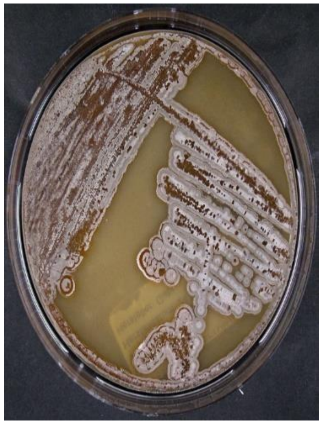<br><br>https://bacdive.dsmz.de/strain/15090	*Streptomyces lannensis* is a source for actinomycin D; a compound responsible for the antagonistic activity against multi-drug resistant pathogens (Dahal *et al.*, 2020).

W44<br><br>soil	*Mesorhizobium albiziae* DSM 21822<br><br>*Mesorhizobium albiziae* CCBAU 61161 (DQ311088)	99.72<br><br>98.42	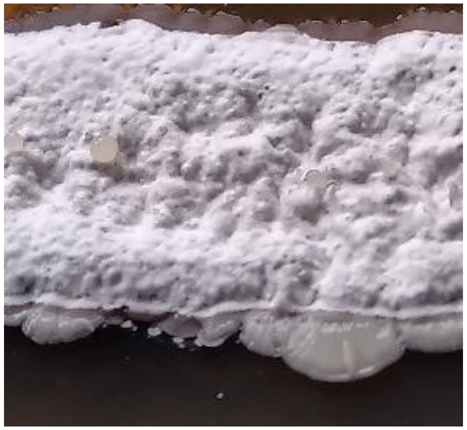	NA	*Mesorhizobium albizae* carries out nitrogen fixation in symbiosis with *Albizia kalkora* (Laranjo *et al.*, 2014).
W48<br><br>soil	*Streptomyces capoamus* JCM 4734<br><br>*Streptomyces viridochromogenes* LS1303-2-23 (KT597548)	99.22<br><br>98.98	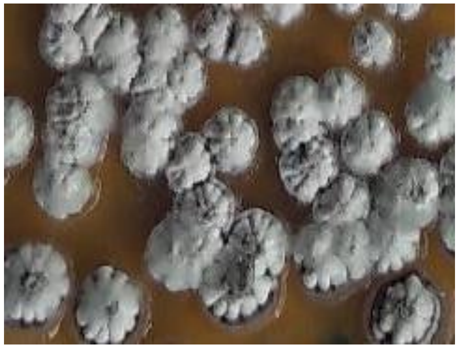	NA	*Streptomyces copoamus* has strong antifungal and antibacterial activity towards various multi-drug resistant pathogens (Singh *et al*., 2007).<br><br>It has various bioactive compounds that are effective against bacterial wilt disease in banana plants (Kawuri & Darmayasa, 2019).<br><br>*Streptomyces viridochromogenes* has the ability to produce Avilamycin; an antimicrobial agent against multi-drug resistant Gram-positive bacteria and is widely used in the human food and animal feed industries (Lv *et al*., 2013).

W51<br><br>soil	*Bacillus wiedmannii* FSL W8-0169<br><br>Bacterium 8-gw1-9 (DQ990037)	99.58<br><br>97.31	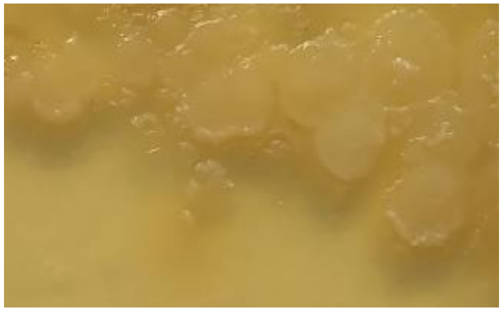	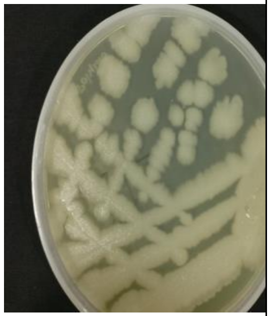	*Bacillus wiedmannii* has been considered an excellent candidate for industrial production of bioplastics from agricultural wastes (Danial *et al.*, 2021).<br><br>It is used in the bioremediation of palladium (Pd) under anaerobic conditions (Chen *et al.*, 2018).

W53<br><br>soil	*Lysinibacillus fusiformis* NBRC 15717<br><br>*Lysinibacillus fusiformis* strain RB-21 (CP010820)	99.52<br><br>99.79	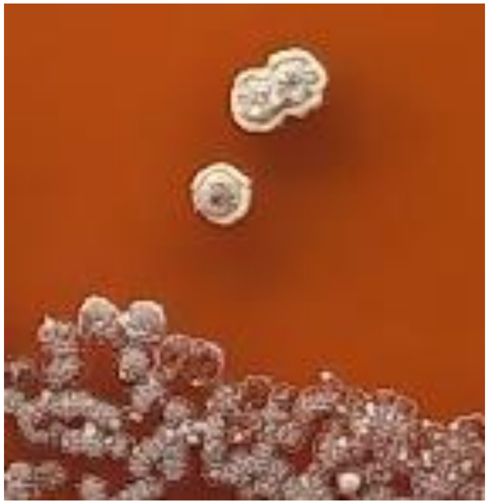		*Lysinibacillus fusiformis* is proficient for the bioremediation of soluble chromate-contaminated soil by reducing its toxicity and bioavailability (Huang *et al*., 2016). It has greater antagonistic activity against antibiotic-resistant pathogens (*Pseudomonas aerugninosa*, *Klebsiella* sp., & *Streptococcus pneumonia*) (Abideen & Babuselvam, 2014) and is further used in the treatment of wastewater rich in metal ions (Taieb *et al*., 2021).
