# Supplementary material for: Isolation, antibacterial screening, and identification of bioactive cave dwelling bacteria in Fiji
Source: Front Microbiol. 2022 Dec 20;13:1012867. doi: 10.3389/fmicb.2022.1012867 (PMC9807670; doi:10.3389/fmicb.2022.1012867)
Supplement: Supplementary file 2 [file Data_Sheet_2.pdf]

References

Abideen, S., & Babuselvam, M. (2014). Antagonistic activity of Lysinibacillus fusiformis n 139 strain isolated from marine fish Triacanthus strigilifer and genome sequence. *International Journal of Current Microbiology and Applied Science*, ***3***(4), 1066– 1072.

Abreo, E., Valle, D., Mujica, V., & Altier, N. (2018). Pathogenicity and virulence factors of Lysinibacillus xylanilyticus and Bacillus spp. towards Argyrotaenia sphaleropa larvae (Lepidoptera). *Journal of Applied Entomology*, ***142***(9), 882–892. https://doi.org/https://doi.org/10.1111/jen.12539

Ahsan, N., Marian, M., Suga, H., & Shimizu, M. (2021). Lysinibacillus xylanilyticus strain GIC41 as a potential plant biostimulant. *Microbes and Environments*, ***36***(4), ME21047. https://doi.org/10.1264/jsme2.ME21047

Almeida, E. L., Carrillo Rincón, A. F., Jackson, S. A., & Dobson, A. D. W. (2019). Comparative genomics of marine sponge-derived Streptomyces spp. isolates SM17 and SM18 with their closest terrestrial relatives provides novel insights into environmental niche adaptations and secondary metabolite biosynthesis potential. *Frontiers in Microbiology*, ***10***, 1713. https://doi.org/https://doi.org/10.3389/fmicb.2019.01713

Bustos, M. C., Ibarra, H., & Dussán, J. (2018). The golden activity of Lysinibacillus sphaericus: new insights on gold accumulation and possible nanoparticles biosynthesis. *Materials*, ***11***(9), 1587. https://doi.org/https://doi.org/10.3390/ma11091587

Cavados, C. de F. G., Pires, E. S., Chaves, J. Q., Alvarez, D. N., Gil, H. B., Oliveira, I. B. R. de, Cunha, A. de B. P. V., & AraújoCoutinho, C. J. P. da C. de. (2017). Isolation and genetic characterization of *Lysinibacillus sphaericus* strains found in mosquito larvae (Diptera: Culicidae). *Research and Reports in Tropical Medicine*, ***8***, 17–20. https://doi.org/https://doi.org/10.2147/RRTM.S124066

Chai, C. H., Hong, C.-F., & Huang, J.-W. (2022). Identification and characterization of a multifunctional biocontrol agent, Streptomyces griseorubiginosus LJS06, against cucumber Anthracnose. *Frontiers in Microbiology*, ***13***, 923276. https://doi.org/10.3389/fmicb.2022.923276

Chen, Y., Chen, Y., Wu, J., & Zhang, J. (2018). The effect of biotic and abiotic environmental factors on Pd(II) adsorption and reduction by Bacillus wiedmannii MSM. *Ecotoxicology and Environmental Safety*, ***162***, 546–553. https://doi.org/https://doi.org/10.1016/j.ecoenv.2018.07.043

Chouhan, R. S., Pandey, A., Qureshi, A., Ozguz, V., & Niazi, J. H. (2016). Nanomaterial resistant microorganism mediated reduction of graphene oxide. *Colloids and Surfaces B: Biointerfaces*, ***146***, 39–46. https://doi.org/https://doi.org/10.1016/j.colsurfb.2016.05.053

Dahal, R. H., Nguyen, T. M., Pandey, R. P., Yamaguchi, T., Sohng, J. K., Noh, J., Myung, S.-W., & Kim, J. (2020). The genome insights of Streptomyces lannensis T1317-0309 reveals actinomycin D production. *The Journal of Antibiotics*, ***73***(12), 837–844. https://doi.org/https://doi.org/10.1038/s41429-020-0343-0

Danial, A. W., Hamdy, S. M., Alrumman, S. A., Gad El-Rab, S. M. F., Shoreit, A. A. M., & Hesham, A. E. (2021). Bioplastic production by Bacillus wiedmannii AS-02 OK576278 using different agricultural wastes. In *Microorganisms* (Vol. 9, Issue 11, p. 2395). https://doi.org/10.3390/microorganisms9112395

Das, G., Patra, J. K., Choi, J., & Baek, K.-H. (2017). Anticandidal effect of endophytic bacteria isolated from Equisetum arvense L.
against Candida albicans and Candida glabrata. *Brazilian Archives of Biology and Technology*, ***60***, 1–16. https://doi.org/https://doi.org/10.1590/1678-4324-2017160433

Donald, L., Pipite, A., Subramani, R., Owen, J., Keyzers, R. A., & Taufa, T. (2022). Streptomyces: still the biggest producer of new natural secondary metabolites, a current perspective. In *Microbiology Research* (Vol. 13, Issue 3, pp. 418–465). https://doi.org/10.3390/microbiolres13030031

El-Tarabily, K. A., AlKhajeh, A. S., Ayyash, M. M., Alnuaimi, L. H., Sham, A., ElBaghdady, K. Z., Tariq, S., & AbuQamar, S. F. (2019). Growth promotion of Salicornia bigelovii by Micromonospora chalcea UAE1, an endophytic 1-aminocyclopropane-1carboxylic acid deaminase-producing actinobacterial isolate. In *Frontiers in Microbiology* (Vol. 10). https://www.frontiersin.org/articles/10.3389/fmicb.2019.01694

Hifnawy, M. S., Fouda, M. M., Sayed, A. M., Mohammed, R., Hassan, H. M., AbouZid, S. F., Rateb, M. E., Keller, A., Adamek, M., & Ziemert, N. (2020). The genus Micromonospora as a model microorganism for bioactive natural product discovery. *RSC Advances*, ***10***(35), 20939–20959. https://doi.org/10.1039/D0RA04025H

Huang, J., Li, J., & Wang, G. (2016). Production of a microcapsule agent of chromate-reducing Lysinibacillus fusiformis ZC1 and its application in remediation of chromate-spiked soil. *SpringerPlus*, ***5***(1), 561. https://doi.org/https://doi.org/10.1186/s40064-0162177-6

Jeong, S.-W., & Kim, J. (2015). Psychrobacillus soli sp. nov., capable of degrading oil, isolated from oil-contaminated soil.
International Journal of Systematic and Evolutionary Microbiology, ***65***(9), 3046–3052. https://doi.org/https://doi.org/10.1099/ijs.0.000375

Kawuri, R., & Darmayasa, I. (2019). Bioactive compound of Streptomyces capoamus as biocontrol of bacterial wilt disease on banana plant. *Earth and Environmental Science*, ***347***(1), 12054. https://doi.org/10.1088/1755-1315/347/1/012054

Laranjo, M., Alexandre, A., & Oliveira, S. (2014). Legume growth-promoting rhizobia: an overview on the Mesorhizobium genus.
Microbiological Research, ***169***(1), 2–17. https://doi.org/https://doi.org/10.1016/j.micres.2013.09.012

Lertcanawanichakul, M., Pondet, K., & Kwantep, J. (2015). In vitro antimicrobial and antioxidant activities of bioactive compounds (secondary metabolites) extracted from Streptomyces lydicus A2. *Journal of Applied Pharmaceutical Science*, ***5***(2), 17–21. https://doi.org/10.7324/JAPS.2015.50204

Lv, X.-A., Jin, Y.-Y., Li, Y.-D., Zhang, H., & Liang, X.-L. (2013). Genome shuffling of Streptomyces viridochromogenes for improved production of avilamycin. *Applied Microbiology and Biotechnology*, ***97***(2), 641–648. https://doi.org/https://doi.org/10.1007/s00253-012-4322-7

Mendes-Silva, T. de C. D., Vidal, E. E., de Souza, R. de F. R., Schmidt, K. da C., Mendes, P. V. D., da Silva Andrade, R. F., da Silva Oliveira, F. G., de Lucena, B. T. L., de Oliveira, M. B. M., dos Santos Correia, M. T., & da Silva, M. V. (2021). Production of carotenoid sarcinaxanthin by Kocuria palustris isolated from Northeastern Brazil Caatinga soil and their antioxidant and photoprotective activities. *Electronic Journal of Biotechnology*, ***53***, 44–53. https://doi.org/https://doi.org/10.1016/j.ejbt.2021.05.004

Nakaew, N., Pathom-aree, W., & Lumyong, S. (2009). Generic diversity of rare actinomycetes from Thai cave soils and their possible use as new bioactive compounds. *Actinomycetologica*, ***23***(2), 21–26. https://doi.org/https://doi.org/10.3209/saj.SAJ230201

Palaniyandi, S. A., Yang, S. H., & Suh, J.-W. (2013). Extracellular proteases from Streptomyces phaeopurpureus ExPro138 inhibit spore adhesion, germination and appressorium formation in Colletotrichum coccodes. *Journal of Applied Microbiology*, ***115***(1), 207–217. https://doi.org/https://doi.org/10.1111/jam.12212

Pan, D., Xu, Y., Ni, Y., Zhang, H., Hua, R., & Wu, X. (2022). The efficient persistence and migration of Cupriavidus gilardii T1 contribute to the removal of MCPA in laboratory and field soils. *Environmental Pollution*, ***304***, 119220. https://doi.org/https://doi.org/10.1016/j.envpol.2022.119220

Pérez, M., Schleissner, C., Fernández, R., Rodríguez, P., Reyes, F., Zuñiga, P., de la Calle, F., & Cuevas, C. (2016). PM100117 and PM100118, new antitumor macrolides produced by a marine Streptomyces caniferus GUA-06-05-006A. *The Journal of Antibiotics*, ***69***(5), 388–394. https://doi.org/https://doi.org/10.1038/ja.2015.121

Raguvaran, K., Kalpana, M., Manimegalai, T., & Maheswaran, R. (2022). Larvicidal, antibacterial, antibiofilm, and anti-quorum sensing activities of silver nanoparticles biosynthesized from Streptomyces sclerotialus culture filtrate. *Materials Letters*, ***316***, 132000. https://doi.org/https://doi.org/10.1016/j.matlet.2022.132000

Riahi, H. S., Heidarieh, P., & Fatahi-Bafghi, M. (2022). Genus Pseudonocardia: what we know about its biological properties, abilities and current application in biotechnology. *Journal of Applied Microbiology*, ***132***(2), 890–906. https://doi.org/https://doi.org/10.1111/jam.15271

Romano, L. H., Granato, A. C., Montenegro, R., Hokka, C. O., Badino-Junior, A. C., & Paiva de Sousa, C. (2014). Marine Streptomyces acrimycini and S. cebimarensis can produce cytotoxic activity on cells HCT-8 and SF295. *Toxicon*, ***91***, 182. https://doi.org/https://doi.org/10.1016/j.toxicon.2014.08.051

Setiawan, A., Setiawan, F., Juliasih, N. L., Widyastuti, W., Laila, A., Setiawan, W. A., Djailani, F. M., Mulyono, M., Hendri, J., & Arai, M. (2022). Fungicide activity of culture extract from Kocuria palustris 19C38A1 against Fusarium oxysporum. In *Journal of Fungi* (Vol. 8, Issue 3, p. 280). https://doi.org/10.3390/jof8030280

Singh, V., Tripathi, C. K. M., & Bihari, V. (2007). Production, optimization and purification of an antifungal compound from Streptomyces capoamus MTCC 8123. *Medicinal Chemistry Research*, ***17***(2), 94. https://doi.org/https://doi.org/10.1007/s00044007-9040-9

Taieb, I., Ben Younes, S., Messai, B., Mnif, S., Mzoughi, R., Bakhrouf, A., Jabeur, C., Ayala Serrano, J. A., & Ellafi, A. (2021). Isolation, characterization and identification of a new Lysinibacillus fusiformis strain ZC from metlaoui phosphate laundries wastewater: bio-treatment assays. *Sustainability*, ***13***(18), 10072. https://doi.org/https://doi.org/10.3390/su131810072

Tedsree, N., Likhitwitayawuid, K., Sritularak, B., & Tanasupawat, S. (2022). Diversity and antimicrobial activity of plant growth promoting endophytic actinomycetes isolated from Thai orchids. *Environment and Natural Resources Journal*, ***20***(4), 379–392. https://doi.org/10.32526/ennrj/20/202200039

Xu, J., Kloepper, J. W., Huang, P., McInroy, J. A., & Hu, C. H. (2018). Isolation and characterization of N2-fixing bacteria from giant reed and switchgrass for plant growth promotion and nutrient uptake. *Journal of Basic Microbiology*, ***58***(5), 459–471. https://doi.org/https://doi.org/10.1002/jobm.201700535

Yasuo, O., Jun, I., Hirofumi, H., Hirokazu, S., Miwa, I., Haruo, I., Atsushi, Y., Masahira, H., & Sueharu, H. (2008). Genome sequence of the streptomycin-producing microorganism Streptomyces griseus IFO 13350. *Journal of Bacteriology*, ***190***(11), 4050–4060. https://doi.org/https://doi.org/10.1128/JB.00204-08
